# Genomic comparison between *Mycobacterium bovis* and *Mycobacterium microti* and *in silico* analysis of peptide-based biomarkers for serodiagnosis

**DOI:** 10.3389/fvets.2024.1446930

**Published:** 2024-09-20

**Authors:** Charlotte Moens, Bert Bogaerts, Victor Lorente-Leal, Kevin Vanneste, Sigrid C. J. De Keersmaecker, Nancy H. C. Roosens, Laurent Mostin, David Fretin, Sylvie Marché

**Affiliations:** ^1^Laboratory of Veterinary Bacteriology, Department of Animal Infectious Diseases, Sciensano, Brussels, Belgium; ^2^Laboratory of Biochemistry and Genetics of Microorganisms, Louvain Institute of Biomolecular Science and Technology, Université Catholique de Louvain, Louvain-la-Neuve, Belgium; ^3^Transversal Activities in Applied Genomics, Sciensano, Brussels, Belgium; ^4^VISAVET Health Surveillance Centre, Complutense University of Madrid, Madrid, Spain; ^5^Experimental Center Machelen, Sciensano, Machelen, Belgium

**Keywords:** *Mycobacterium microti*, *Mycobacterium bovis*, tuberculosis, B-cell epitopes, diagnosis, whole-genome sequencing, antigens

## Abstract

In recent years, there has been an increase in the number of reported cases of *Mycobacterium microti* infection in various animals, which can interfere with the ante-mortem diagnosis of animal tuberculosis caused by *Mycobacterium bovis*. In this study, whole genome sequencing (WGS) was used to search for protein-coding genes to distinguish *M. microti* from *M. bovis*. In addition, the population structure of the available *M. microti* genomic WGS datasets is described, including three novel Belgian isolates from infections in alpacas. Candidate genes were identified by examining the presence of the regions of difference and by a pan-genome analysis of the available WGS data. A total of 80 genes showed presence-absence variation between the two species, including genes encoding Proline-Glutamate (PE), Proline-Proline-Glutamate (PPE), and Polymorphic GC-Rich Sequence (PE-PGRS) proteins involved in virulence and host interaction. Filtering based on predicted subcellular localization, sequence homology and predicted antigenicity resulted in 28 proteins out of 80 that were predicted to be potential antigens. As synthetic peptides are less costly and variable than recombinant proteins, an *in silico* approach was performed to identify linear and discontinuous B-cell epitopes in the selected proteins. From the 28 proteins, 157 B-cell epitope-based peptides were identified that discriminated between *M. bovis* and *M. microti* species. Although confirmation by *in vitro* testing is still required, these candidate synthetic peptides containing B-cell epitopes could potentially be used in serological tests to differentiate cases of *M. bovis* from *M. microti* infection, thus reducing misdiagnosis in animal tuberculosis surveillance.

## Introduction

Animal tuberculosis (TB) is a chronic infectious disease caused by members of the *Mycobacterium tuberculosis* complex (MTBC), whose natural hosts are wild and domestic mammals (1). *Mycobacterium bovis* is the primary cause of TB in cattle (bovine TB, bTB) and is capable of infecting a wide range of other animal species as well as humans (2). This zoonotic pathogen therefore poses a high risk to animal and human health and to international trade in domestic animals and animal products, highlighting the need for surveillance.

The first reported case of *M. bovis* infection on an alpaca farm in Belgium occurred in 2015 (personal communication from M. Mori, Sciensano, Belgium), increasing the interest in *ante-mortem* diagnostics in New World Camelids (NWCs), such as llamas and alpacas. Diagnostic tests for animal TB detection based on cell-mediated immunity (CMI), such as the intradermal tuberculin test or the IFNɤ assay, have been reported to have poor accuracy in NWCs (3, 4). Serological diagnostic assays may be a promising alternative, as shown by (5). Over the last decade, several serological assays have been developed to detect animal TB in camelids, such as the ELISA INgezim Tuberculosis DR multispecies test (Ingenasa, Spain) and the multiplexed Enferplex Camelids TB test (Enfer Scientific, Ireland) (6, 7).

In 2020, alpacas from two different Belgian farms, one of which was involved in the 2015 animal TB outbreak, were suspected of being infected with *M. bovis* based on positive serological diagnostic results (INgezim Tuberculosis DR multispecies and Enferplex Camelids TB). Necropsy of these animals revealed lesions typical of tuberculosis, and the real-time PCR (qPCR), performed on the organs and using the IS*6110* insertion sequence for identifying MTBC members, was positive. However, the qPCR test to differentiate the MTBC members, based on polymorphisms of the *gyrB* gene and performed from bacterial culture (8), identified *M. microti* (data from the National Reference Laboratory for Bovine Tuberculosis, Sciensano, Belgium).

First identified in voles, *M. microti* is another, less virulent, member of MTBC that can infect a wide range of animal species and cause tuberculous lesions (9–11). Furthermore, the impact of the epidemiological situation of *M. microti* in some areas does not seem to be irrelevant (11–13). However, the European Animal Health Law (EU 2016/429) does not include *M. microti* as an etiological agent of animal TB, which according to the legislation only includes *M. bovis, M. caprae* and *M. tuberculosis*. Consequently, *M. microti* infections are non-notifiable and do not lead to the same sanitary measures (e.g., removed animals, blocked farms) as *M. bovis* or *M. caprae* outbreak declarations. *M. microti*, therefore, represents a risk of interference in the animal TB diagnosis.

Research into biomarkers to accurately distinguish *M. bovis* from *M. microti* infection at *ante-mortem* examination in animals has received limited attention. While the serological tests used to diagnose animal TB in NWCs and other species, such as cattle, are effective in detecting infected animals, they are not specific enough to distinguish between *M. bovis* and *M. microti* infection (14–16). It is therefore important to identify specific antigenic proteins that can elicit an antibody response and that can be used in serological tests. B-cell epitopes are segments of antigenic proteins that can be recognized by antibodies (17), and are categorized into linear (i.e., continuous amino acid sequences) and conformational (i.e., folded three-dimensional structures) epitopes. Of particular interest is the identification of specific B-cell epitopes that could be used to construct synthetic peptides as biomarkers.

Members of the MTBC have over 99.9% DNA sequence identity to each other, but they differ in phenotype, virulence and host species. Differences between species are mostly limited to deletions of regions of difference (RDs), single nucleotide polymorphisms (SNPs) in protein-coding genes and hypervariable regions. Molecular typing of MTBC members is often performed using spoligotyping, a polymerase chain reaction-based method that examines the deletion of spacer sequences in the Direct Repeat (DR) region (18). The resulting spoligotype profiles can be used to understand the genetic diversity and relatedness of MTBC strains. Diverse spoligotypes were reported for *M. microti* strains (11) among them the more dominant are the “llama-type” and “vole-type” spoligotypes, named after the host animals in which they were first observed (19). Studies have shown that *M. microti* strains are characterized by *M. microti*-specific deletion regions (MiD) together with the deletion of part of the RD1 region (RD1^mic^), which includes open reading frames (ORF) of known virulence genes (20). These genomic variations may be useful for the epidemiological surveillance and diagnosis of animal TB (21–23). The genomic organization of members of the MTBC, mainly *M. bovis* and *M. tuberculosis*, has been further explored using whole-genome sequencing (WGS), allowing a better understanding of the pathology and the identification of biomarkers for the diagnosis and management of TB (24–27). However, to date, there has been very little research on *M. microti*, and very few WGS datasets are currently available in public databases.

This study was initiated to identify potential biomarkers for distinguishing *M. microti* from *M. bovis* using WGS. The genomic information was used to screen for variations affecting proteins that could form the basis of serological diagnostic methods. An *in silico* approach was used to extract B-cell epitopes from the identified putative antigenic proteins. In addition, this study describes the genomic diversity of *M. microti* based on publicly available data, including the recently collected strains from Belgium.

## Materials and methods

### Bacterial strains and DNA extraction

Three *M. microti* strains were isolated from the organs of alpacas from Belgian farms. Two were isolated on the same farm from animals with a positive result in serological tests for animal TB (i.e., isolates MI20-1 and MI20-2). Both alpacas showed tuberculous lesions at necropsy. The third isolate was collected from an alpaca that was found dead (with tuberculous lesions) and originating from another farm (i.e., isolate VAR696). There was no apparent geographical or epidemiological link between the two farms. All samples were collected in 2020.

*M. microti* strains MI20-1 and MI20-2 were grown either on a solid stone-brink medium or using a BACTEC™ MGIT™ automated liquid system (Becton Dickinson, Sparks, MD, USA) at the European Reference Laboratory for bovine tuberculosis at the VISAVET Health Center (Madrid, Spain) and incubated at 37°C for several weeks. *M. microti* VAR696 was processed in a similar manner at the Belgian Reference Laboratory for bovine tuberculosis at the Belgian Institute of Health (Sciensano, Brussels, Belgium) and genomic DNA was extracted using the QIAGEN DNeasy Blood and Tissue kit (Qiagen, Hilden, Germany) according to the manufacturer's instructions for Gram-positive bacteria with a prior mechanical grinding step to meet the specific requirements for DNA extraction in the case of *Mycobacteria* spp. Genomic DNA of strains MI20-1 and MI20-2 was extracted from a pure culture grown in Middlebrook 7H9 medium supplemented with oleic albumin dextrose catalase (OADC) using phenol-chloroform-isoamyl alcohol (PCI) and was then purified using ethanol, as previously described by (28). The resulting DNA pellet was dried at room temperature and re-suspended in 50–200 μL of ultrapure distilled water (Sigma-Aldrich, St. Louis, MO, USA).

DNA quality and concentration were measured by spectrophotometry using the Nanodrop^®^ 2000 (Thermo Fisher Scientific, Waltham, MA, USA) and DNA purity was assessed using the A260/A280 and A260/A230 ratios.

Detection of members of the MTBC was performed by a qPCR based on the IS*6110* element (29). The molecular characterization of MTBC mycobacteria was performed using a qPCR based on polymorphism of the *gyrB* gene (8) and/or direct variable repeat (DVR)-spoligotyping (30).

### Whole-genome sequencing

The DNA extracts from isolates MI20-1 and MI20-2 were sent to the National Veterinary Services Laboratory in Ames, Iowa (USA) for library preparation and whole genome sequencing, whereas the VAR696 isolate was sequenced at Sciensano (Belgium). Library preparation was achieved using the Nextera XT library preparation kit (Illumina, San Diego, CA, USA) according to manufacturer's instructions. Sequencing was carried out in both cases on a MiSeq system with the V3 chemistry, obtaining 250 bp paired-end reads, aiming for a theoretical coverage of 60X based on the expected genome size of ~ 4.4 Mbp of *M. microti*.

### Pre-processing and *de novo* assembly

**Figure 1:**
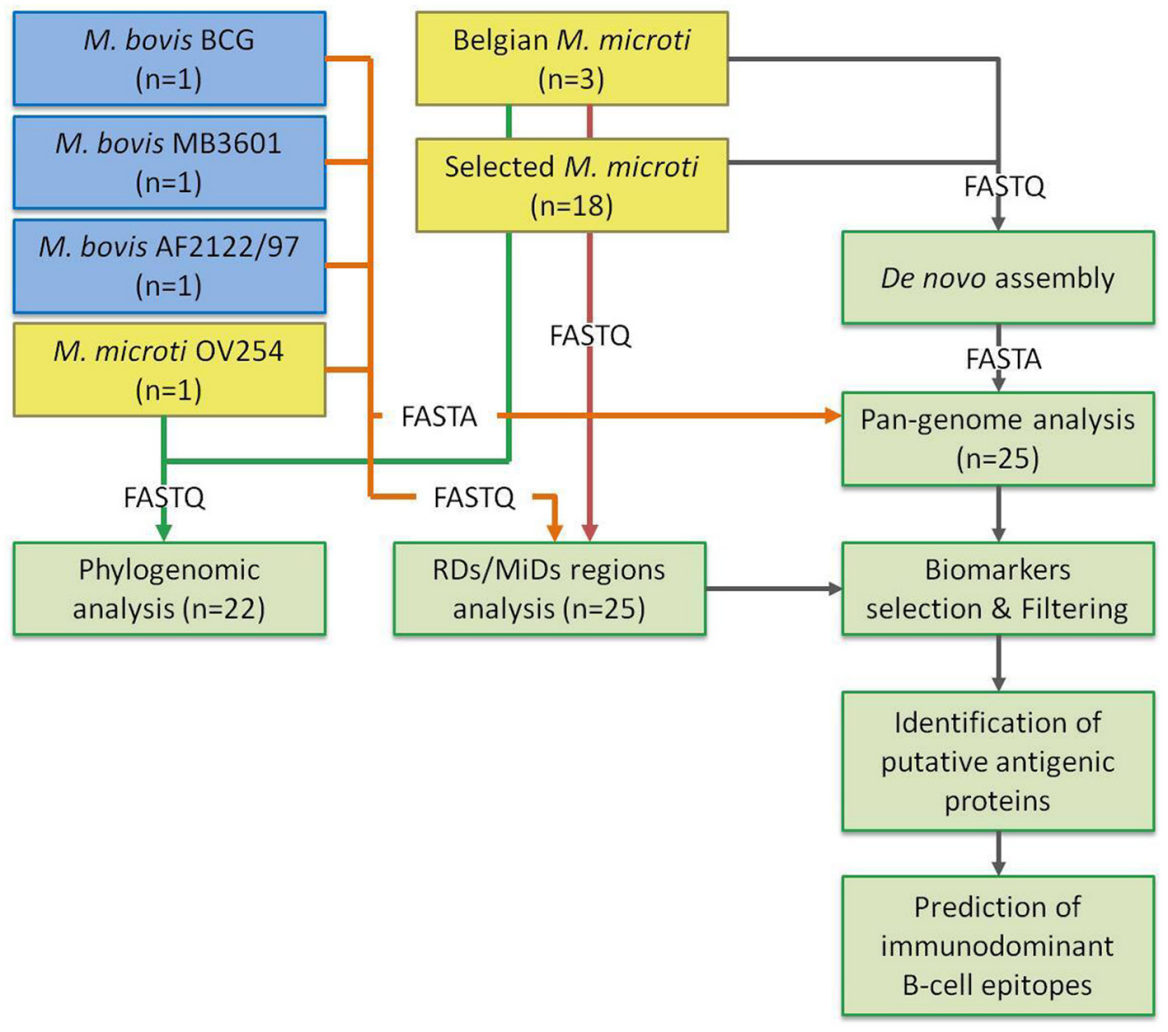
Overview of the bioinformatics processing. Overview of the study design. *M. bovis* and *M. microti* data are shown in blue and yellow boxes, respectively. The number of strains is indicated. The parts of the analysis are shown in green boxes. The arrows indicate the flow of the data, with the format indicated by the label. The green arrow shows the input FASTQ files for the phylogenomic analysis. The orange arrow indicates the data for the selected reference genomes: (1) the FASTQ files are used for the RD/MiD analysis, (2) the genome FASTA files are used as input for the pan-genome analysis. The red arrow indicates the FASTQ input of the Belgian and selected *M. microti* strains for the RD/MiD analysis. The gray arrow indicates the flow of data between the sub-analyses.

Pre-processing and *de novo* assembly were performed as previously described (31). Briefly, raw reads were trimmed using Trimmomatic 0.38 (32) with the following options: “LEADING” set to 10, “TRAILING” set to 10, “SLIDINGWINDOW” set to “4:20,” “MINLEN” set to 40, and “ILLUMINACLIP” set to “NexteraPE-PE.fa:2:30:10.” Processed reads were then assembled *de novo* using SPAdes 3.13.0 (33) with the “—careful” option enabled and “–cov-cutoff” set to 10. Contigs smaller than 1,000 bp were removed using the “seq” function of Seqtk 1.3 (available at https://github.com/lh3/seqtk). The quality of the assemblies was evaluated using QUAST 4.4 (34), with the filtered assemblies as input. Several additional quality checks included in the workflow were also evaluated: (1) screening for contaminants using Kraken 2 v2.0.7 (35); (2) evaluation of median sequencing depth and mapping rate using Bowtie 2 v2.4.1 (36) and SAMtools v1.9 (37); and (3) various quality checks on the input reads with FastQC v0.11.7 (https://www.bioinformatics.babraham.ac.uk/projects/fastqc/). Figure 1 illustrates the flow of the full bioinformatics analysis.

### Phylogenomic analysis

All available *M. microti* datasets in the Sequence Read Archive (SRA) at the National Center for Biotechnology Information (NCBI) that were generated by Illumina sequencing were retrieved (accessed on the 24th of January 2022), for which an overview is provided in Table 1. Three datasets were available for isolate 8753/00, of which ERR551113 was selected because it had the highest coverage. The ERR553377 dataset was also excluded from the analysis as it clustered identically in the SNP analysis with ERR553376, which was collected in the same study. Similarly, the ERR2659165 dataset was excluded from analysis as it is a replicate of the ERR2659166 dataset. Datasets with an estimated coverage >100x, calculated based on the number of bases in the FASTQ files and the genome size of the *M. microti* OV254 genome (RefSeq accession GCF_904810325.1), were downsampled to ~100x using the “sample” function of seqtk 1.3. The SNP phylogeny was then performed using PACU 0.0.5 (44), which uses BCFtools 1.17 (45) for variant calling and Gubbins 3.3.1 (46) to remove SNPs in recombinant regions. First, the processed reads were mapped to the *M. microti* OV254 genome (RefSeq accession GCF_904810325.1), using the PACU_map function with the “–read-type” parameter set to “illumine” and other options left at their default values. The resulting BAM files were used as input for the PACU SNP workflow. The online PHASTER tool (47) was used to generate a BED file with (pro-) phage regions in the reference genome and provided to PACU with the “–ref-bed” option. The –use-mega' option was enabled to perform automatic model selection and tree building with MEGA X (48). The best-fit model was selected as the substitution model with the lowest Bayesian information criterion (BIC) value, selecting the Tamura 3-parameter model. Spoligotypes were determined *in silico* using SpoTyping 2.1 (49) and added as annotations to the phylogeny. The “—min” and “—rmin” parameter values were set to 1 for datasets with an estimated coverage of < 30x, estimated based on the mapping to the reference genome. For other datasets, the default parameter values were used.

**Table 1:** *M. microti* strains used in the phylogenomic analysis.

***M. microti* strain**	**Host**	**Country**	**SRA accession(s)**	**Quality^a^**	**References**
OV254	Vole	UK	ERR027294	OK	(38, 39)
ATCC 35782	Vole	n/a	ERR027295	OK	(20, 39)
ATCC 19422	Vole	UK	SRR3647357	OK	(38)
Maus III (6740/00 or B3)	Human	n/a	ERR4618952	OK	(20, 39)
Maus IV (1479/99 or B4)	Human	n/a	ERR027297	OK	(20, 39)
94-2272	Human	NL	ERR027298	OK	(39, 40)
8753/00	Human	DE	ERR551111	–^b^	
			ERR551112	-^b^	(41) Miru-vntrplus.org
			ERR551113	OK	
416-01	Human	DE	ERR553376	OK	(41)
			ERR553377	-^c^	
G25821	Wild Boar	IT	ERR2659163	OK	(42, 43)
G25822	Wild Boar	IT	ERR2659164	OK	(42, 43)
G25823	Wild Boar	IT	ERR2659165	-^d^	(42, 43)
			ERR2659166	OK	
G25824	Wild Boar	IT	ERR2659167	OK	(42, 43)
G25825	Wild Boar	IT	ERR2659168	OK	(42, 43)
G25826	Wild Boar	IT	ERR2659169	OK	(42, 43)
G25827	Wild Boar	IT	ERR2659170	OK	(42, 43)
G25828	Wild Boar	IT	ERR2659171	OK	(42, 43)
*M. microti*−36	Domestic cat	UK	ERR4627394	OK	APHA
*M. microti*−45	Pig	UK	ERR4627395	OK	APHA
*M. microti*−68	Domestic cat	UK	ERR4627396	OK	APHA
MI20-1	Alpaca	BE	SRR25473321	OK	This study
MI20-2	Alpaca	BE	SRR25473320	OK	This study
VAR696	Alpaca	BE	SRR25473322	OK	This study

^a^ERR027293, ERR027296, ERR3261389, and SRR2667442 not covered *M. microti* genomes.

^b^Not used, replicate of ERR551113.

^c^Not used, replicate of ERR553376.

^d^Not used, replicate of ERR2659166.

n/a, not available; UK, United Kingdom; NL, The Netherlands; DE, Germany; IT, Italy; BE, Belgium; APHA, Animal and Plant Health Agency.

### Genomic characterization of RDs and MiDs regions

The presence of RDs and MiDs was assessed using a read mapping-based approach. The RD and MiD nomenclature used in this study is based on that of (20, 21, 50–52) and differs from that proposed by (53). An overview of the evaluated RDs and MiDs is provided in Table 2. Bowtie2 2.4.1 was used with the “–very-sensitive-local” preset enabled to map the processed reads to the sequences of the RD regions, obtained from the *M. tuberculosis* H37Rv reference genome (RefSeq accession NC_000962.3). The analysis included the Illumina FASTQ datasets for the *M. bovis* BCG Danish 1311 (SRR7983756), *M. bovis* AF2122/97 (ERR1744454), *M. bovis* MB3601 (ERR3825346), and *M. microti* OV254 (ERR027295) reference genomes and all *M. microti* strains included in the phylogenomic analysis. Regions that were covered for at least 75% and with a median depth >5x were considered to be present.

**Table 2:** Overview of the evaluated RDs and MiDs.

**Region**	**Loci^a^**	**Position^b^**	**Length (bp)**	***M. bovis* AF2122/97 and MB3601 (virulent strains)**	***M. bovis* BCG (vaccine strain)**	**All *M. microti* strains**
MiD1	Rv2816c-Rv2818c	3123603–3126144	2,541	+	+	+/-
MiD2	Rv3188-Rv3189	3554287–3555262	975	+	+	+/-
MiD3	Rv3345c-Rv3349c	3738073–3755237	17,164	+	+	-
N-RD25^bov/cap^	Rv3738c-Rv3740c	4189270–4192179	2,909	-	-	+
Pks15/1	Rv2946c-Rv2947c	3291464–3297840	6,376	+	+	+
RD10	Rv0221-Rv0223c	264040–267764	3,724	-	-	-
RD105	Rv0071-Rv0074	79447–83983	4,536	+	+	+
RD11	Rv2645-Rv2659c	2970067–2980818	10,751	-	-	+
RD115	Rv0376c-Rv0378	453173–455868	2,695	+	+	+
RD122	Rv0576	669840–671154	1,314	+	+	+
RD12^bov^	Rv3117-Rv3121	3483957–3487711	3,754	-	-	+
RD13	Rv1255c-Rv1257c	1402761–1406084	3,323	-	-	+
RD14	Rv1765c-Rv1773c	1997380–2007766	10,386	+	+	+
RD142	Rv1189-Rv1192	1332039–1335754	3,715	+	+	+
RD149	Rv1585c-Rv1587c	1786552–1789163	2,611	+	-	-
RD15	Rv0309-Rv0312c	377903–382418	4,515	+	+	+
RD150	Rv1671-Rv1674c	1896474–1899916	3,442	+	+	+
RD16	Rv3400-Rv3405c	3817212–3825268	8,056	+	+	+
RD174	Rv1992c-Rv1997	2234938–2242876	7,938	+	+	+
RD182	Rv2270-Rv2280	2544674–2552939	8,265	+	+	+
RD183	Rv2313-Rv2315c	2585024–2588812	3,788	+	+	+
RD193	Rv2406c-Rv2407	2703981–2705518	1,537	+	+	+
RD1^bcg^	Rv3871-Rv3879	4348741–4359782	11,041	+	-	-
RD1^mic^	Rv3864-Rv3876	4340184–4355010	14,826	+	-	-
RD2	Rv1978-Rv1988	2220887–2232219	11,332	+	-	+
RD207	Rv2814c-Rv2820c	3119061–3128272	9,211	+	+	+/-
RD219	Rv3083-Rv3085	3448447–3451750	3,303	+	+	+
RD239	Rv3651	4091823–4092880	1,057	+	+	+
RD3	Rv1573-Rv1586c	1779291–1788505	9,214	+	-	-
RD4	Rv1505c-Rv1516c	1695239–1708539	13,300	-	-	+
RD5^mic^	Rv2349c-Rv2353c	2627085–2635599	8,514	-	-	+/-
RD6	Rv3425-Rv3428c	3842202–3845970	3,768	-	-	+/-
RD7	Rv1964-Rv1977	2207644–2220800	13,156	-	-	-
RD702	Rv0186	216260–218344	2,084	+	+	+
RD711	Rv1333-Rv1336	1501577–1504365	2,788	+	+	+
RD724	Rv2018-Rv2019	2265261–2266419	1,158	+	+	+/-
RD726	Rv3485c-Rv3487c	3904619–3907007	2,388	+	+	+
RD750	Rv1519-Rv1520	1710729–1712070	1,341	+	+	+
RD8	Rv3617-Rv3623c	4057714–4063249	5,535	-	-	-
RD9	Rv2072c-Rv2075	2328939–2332879	3,940	-	-	-

This table lists the regions of difference (RDs) and *M. microti*-specific deletion regions (MiDs). Note that some regions may be (partially) overlapping. bp, base pair. The last three columns indicate whether the corresponding region was present (+), absent (-), or variable (+/-) in the respective strains.

^a^The loci column refers to loci from the *M. tuberculosis* H37Rv genome annotation.

^b^The position column refers to the position within the *M. tuberculosis* H37Rv genome.

### Pan-genome analysis

Roary was used for the pan-genomic analysis. Three *M. bovis* reference strains (i.e., the *M. bovis* BCG vaccine strain and the two virulent *M. bovis* strains AF2122/97 and MB3601) and one *M. microti* reference strain (OV254) were used, for which the circular genomes were downloaded from NCBI RefSeq. In addition, the assembled genomes of the *M. microti* strains used in the phylogenomic analysis were also used, including those of the three Belgian strains. Prokka 1.14.6 (54) was used with default options for gene prediction and functional annotation. The proteins from the *M. tuberculosis* H37Rv, *M. microti* OV254, *M. bovis* AF2122/97, and *M. bovis* BCG Danish 1311 reference genomes were provided with the “—proteins” options as a basis for gene annotation. Roary 3.12.0 (55) was then used for the pan-genome analysis with the “—mafft” option enabled, minimum identity set to 90%, and paralogs not split (“-s”). UpSet plots were generated using the UpSetPlot python package v0.8.0 (available at https://github.com/jnothman/UpSetPlot) to show the overlap of genes between species and strains. The results of the pan genome analysis were used to identify protein-encoding genes that could be used as potential biomarkers for the differentiation of *M. microti* from virulent *M. bovis* (i.e., excluding the *M. bovis* Danish 1311 BCG vaccine strain).

### Selection of potential biomarkers

Proteins, from the RD/MiDs and pan-genome analyses, present in the virulent *M. bovis* strains (i.e., not the *M. bovis* BCG vaccine strain) and absent in all *M. microti* strains, and vice-versa, were selected as potential biomarkers. They were then evaluated according to the following three criteria: subcellular localization, sequence homology and antigenicity, as elaborated below.

#### Prediction of subcellular localization

SignalP 6.0 was used to predict the presence of sec- and tat-dependent signal peptides in proteins (56). These short N-terminal amino acid sequences are recognized by the classical sec or tat translocation machinery and thus control protein secretion and translocation in bacteria. SecretomeP 2.0 was used for the prediction of non-classical secreted proteins (57). Both programs are freely available on the website of the Center for Biological Sequence Analysis at the Technical University of Denmark (http://www.cbs.dtu.dk/services). In addition, Psortb v3.0.3 [(58); https://www.psort.org/psortb/], Gpos-mPLoc [(59); http://www.csbio.sjtu.edu.cn/bioinf/Gpos-multi/] and CELLO2.5 [(60); http://cello.life.nctu.edu.tw/] were used for predictions of the subcellular localization of the candidate proteins. CELLO2.5 is a multi-class Support vector machine (SVM) classification system using four types of sequence coding schemes. Psortb3.0.3 uses a combination of modules, each of which is known to influence subcellular location of the proteins (e.g., the number of transmembrane helices, signal peptides, outermembrane motifs, ...). Gpos-mPLoc identifies subcellular localization of Gram positive bacterial proteins by fusing the information of gene ontology, as well as the functional domain information and sequential evolution information. Amino acid sequences were retrieved from the NCBI protein database and used as input for all tools. The UniProt database (http://www.uniprot.org/) was also queried to obtain the subcellular localization of the proteins.

#### Sequence homology analysis

Putative biomarkers with high sequence similarity to proteins from members of the *Mycobacterium avium-intracellulare* complex (MAC; *M. avium* subspecies *paratuberculosis*, “*M. avium* subspecies *hominissuis*,” *M. avium* subspecies *avium*, and *M. intracellulare*) were removed. These environmental mycobacteria are commonly isolated from cattle and share proteins with members of MTBC that can cross-react in diagnosis (61–63). Sequence similarities of all proteins were evaluated using the non-redundant protein sequence database of the NCBI BLASTp tool (https://blast.ncbi.nlm.nih.gov/). Putative proteins with percent identity ≥55% and query coverage ≥40% to those in the NCBI database were discarded.

#### Prediction of antigenicity

The antigenicity of all proteins was estimated using ANTIGENpro (http://scratch.proteomics.ics.uci.edu/) and VaxiJen v2.0 (https://www.ddg-pharmfac.net/vaxijen/VaxiJen/VaxiJen.html). The predictions from ANTIGENpro are based on a two-step approach, combining multiple representations of the primary sequence and machine learning algorithms. A final support vector machine (SVM) classifier summarizes the resulting predictions and provides a probability of whether the protein is likely to be antigenic (64). VaxiJen 2.0 is an alignment-free approach for antigen prediction, which is based on auto cross covariance transformation of protein sequences into uniform vectors of key amino acid properties (65). A prediction threshold of 0.5 was used for both programs, as recommended in literature (64, 65). Proteins predicted to be antigenic by at least one of these two tools were considered likely to be antigenic.

### Prediction of linear and conformational B-cell epitopes

The prediction of linear and conformational B-cell epitopes was performed for all proteins predicted as extracellular, low conserved in environmental mycobacteria and antigenic.

For the prediction of linear B-cell epitopes, protein sequences in FASTA format were submitted to five different tools available in the Epitope Database and Analysis Resource (IEDB; https://www.iedb.org/), based on the following scales: beta turn (66), surface accessibility (67), flexibility (68), hydrophilicity (69), and antigenicity (70). For each tool, the window size was set to seven amino acids and the default threshold was used. BepiPred-2.0, available on the IEDB server, was also used to predict linear B-cell epitopes from amino acid sequences in FASTA format. The Bepipred 2.0 tool is based on a random forest algorithm trained on epitope and non-epitope amino acids as determined from crystal structures. The default threshold value of 0.50 was used (71). All results (i.e., all linear B-cell epitopes predicted) obtained with the IEDB tools were compared to find similar epitopes using the Epitope Cluster Analysis v2.0 tool (http://tools.iedb.org/cluster/), with a minimum sequence identity threshold of 70%. The consensus epitopes obtained with the Epitope Cluster tool were selected as the final linear B-cell epitopes. Prediction of linear B-cell epitopes via multiple tools and peptides clustering was limited for protein sequences with a high number of amino acids (>1,500), meanly PE_PGRS50 (Rv3345c) and PPE55 (Rv3347c). Consequently, only antigenic linear B-cell epitopes predicted by the Kolaskar and Tongaonkar method were used for these proteins.

As most of B-cell Epitopes in native proteins are conformational (72), the conformational B-cell epitopes were predicted by using the Discotope 3.0 (https://services.healthtech.dtu.dk/services/DiscoTope-3.0/) and ElliPro http://tools.iedb.org/ellipro/; (73) servers. Proteins structures were downloaded from the PDB database (https://www.rcsb.org/) or the AlphaFold protein structure database [https://alphafold.ebi.ac.uk/; (74)]. The default parameters were used. Amino acid sequences, ranging from 8 to 25 residues, that were common to the predicted B-cell linear and conformational epitopes, were retained. The antigenicity of B-cell Epitopes predicted as linear and conformational was analyzed with Vaxijen2.0. The epitopes with a value >0.5 were considered as the best B-cell epitope peptides.

### Data availability

The datasets supporting the conclusions of this study have been deposited in the NCBI SRA under accession number PRJNA1000766. Individual accession numbers are provided in Table 1.

## Results

### Pre-processing and data quality

Data quality metrics for the *de novo* assembly and read mapping analysis of the in-house generated datasets (i.e., the Belgian *M. microti* isolates) are provided in Supplementary Table 1. All three datasets passed the quality checks of the workflow (31), indicating that the data were suitable for further analysis.

### Phylogenomic analysis of available *M. microti* genomes

**Figure 2:**
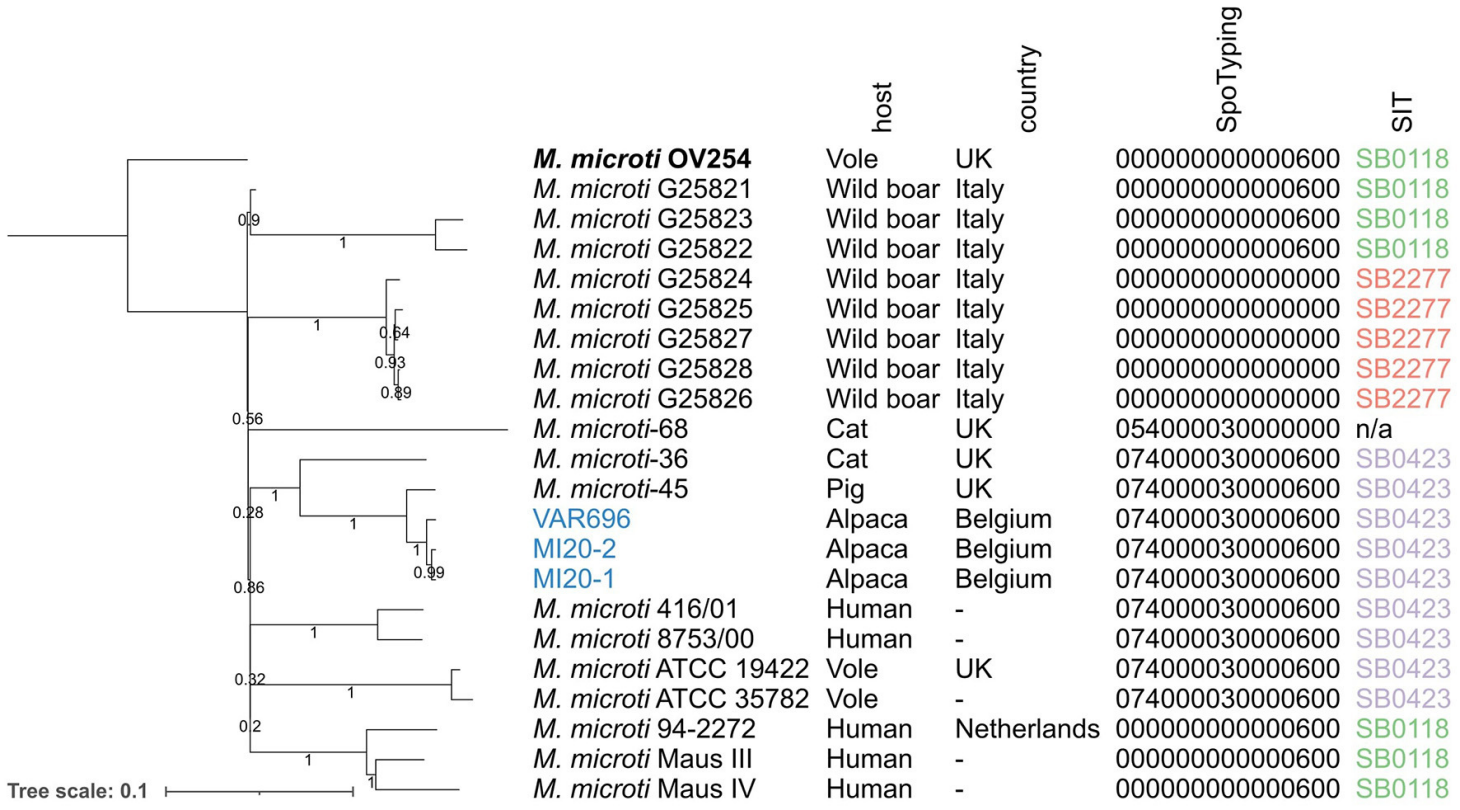
Maximum likelihood phylogeny of the *M. microti* strains collected from NCBI and the isolates collected in this study. Branch lengths and the scale bar are expressed as average substitutions per site. Bootstrap support is indicated by the numbers on the branches (proportions based on 1,000 replicates). *M. microti* OV254 was used as the reference genome for the phylogeny (highlighted in bold). The three isolates collected as part of this study are shown in blue. Annotations are (from left to right): strain name, host species, country, spoligotype determined by SpoTyping in octal format, and SIT number. The phylogeny was mid-point rooted. UK, United Kingdom; n/a, not available; SIT, Spoligotype International Type.

Of the 27 *M. microti* datasets collected from NCBI, 19 were included in the phylogenomic analysis, with eight excluded due to low quality. These data were supplemented by the three *M. microti* strains sequenced in this study, resulting in a total of 22 *M. microti* strains (Table 1). The output of the maximum likelihood analysis is shown in Figure 2 and pairwise SNP distances are shown in Supplementary Table 2. The isolates collected in this study show very high genomic similarity, with only eight SNP differences between MI20-1 and MI20-2, and 18 SNPs between these two isolates and VAR696. However, a direct epidemiological link between these strains seems unlikely given the low mutation rate of MTBC (75). The closest strain collected from SRA was *M. microti*-45, isolated from a pig in the United Kingdom (UK), which differed by 57 SNPs from all three Belgian strains in this study. The *M. microti*-36 strain, isolated from a domestic cat in the UK, showed 241–243 SNP differences with the three Belgian strains and 244 SNP differences with *M. microti*-45. While the bootstrap values for the longer branches were generally high, the bootstrap values also indicated low confidence in the relative position of these branches.

The three Belgian strains were assigned to the same spoligotype as the *M. microti-*45 and *M. microti*-36 strains in the same clade (the “llama-type” spoligotype SB0423). The same spoligotype was observed in two other clades: (1) the clade carrying *M. microti* ATCC 19422 and ATCC35782, collected from voles, and (2) the clade carrying *M. microti* 416/01 and 8753/00, both isolated from humans. The *M. microti*-68 strain had a similar spacer pattern, but lacked spacers 37 and 38, resulting in a different spoligotype. All other isolates were assigned to SB0118, known as the “vole-type” spoligotype, except for the clade containing five isolates collected from wild boars in Italy, which lacked all spacers, assigning them to SB2277.

### Genomic characterization of RDs and MiDs regions

**Figure 3:**
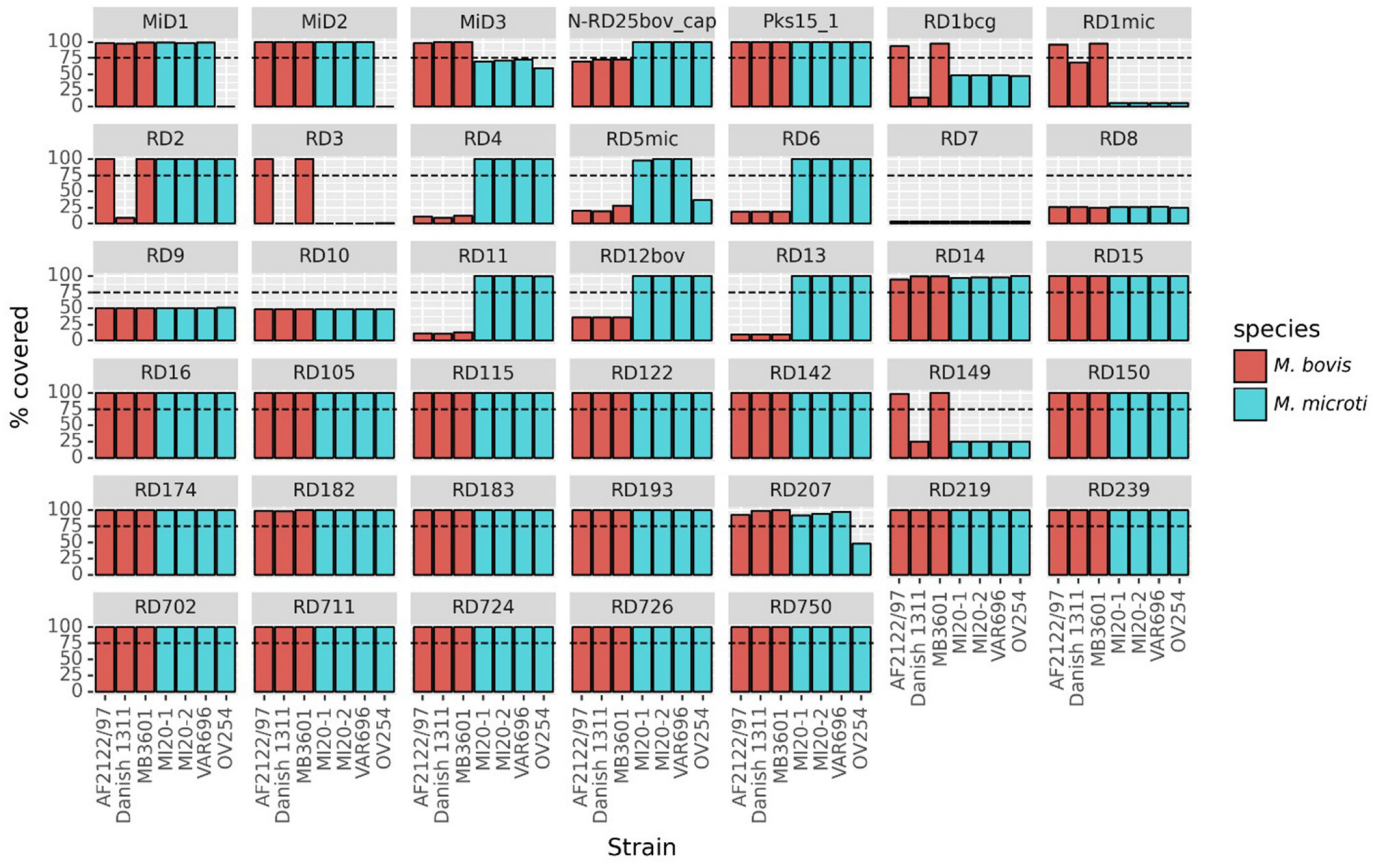
Presence of RDs and MiDs regions in the Belgian *M. microti* datasets and the selected reference genomes. This graph shows the coverage percentage of the regions of difference (RDs) and *M. microti*-specific deleted regions (MiDs). The y-axis indicates the percentage of the region that was covered by reads, the x-axis indicates the different strains. The bars are colored by species as indicated in the legend. The dashed line indicates the 75% threshold that was used to classify regions as present or absent. The following reference and Belgian strains were included: *M. bovis* BCG Danish 1311 (SRR7983756), *M. bovis* AF2122/97 (ERR1744454), *M. bovis* MB3601 (ERR3825346), *M. microti* OV254 (ERR027295), *M. microti* VAR696 (SRR25473322), *M. microti* MI20-1 (SRR25473321), and *M. microti* MI20-2 (SRR25473320).

Twenty out of the 40 RDs/MiDs regions analyzed were present in the three Belgian and the OV254 reference *M. microti* strains along with in the three *M. bovis* reference strains studied, as shown in Figure 3. The RD7, RD8, RD9, and RD10 regions were (mostly) absent in all strains, except for *M. tuberculosis* H37Rv. The RD4, RD6, RD11 (prophage phiRv2), RD12^bov^, RD13, and N-RD25^bov/cap^ regions were absent in all *M. bovis* strains (virulent and vaccine strains), but present in the four *M. microti* strains. However, ~60% (below the detection threshold of 75%) of the N-RD25^bov/cap^ region was present in the *M. bovis* strains, with the PPE66 (Rv3738c) ORF interrupted and the PPE67 (Rv3739c) locus completely absent in these strains. No intact ORFs were found in the fractions (< 25% coverage) of the RD4, RD6, RD11, and RD13 regions that were present in the *M. bovis* strains. Similarly, no intact ORFs were found for the fraction of the RD12^bov^ region (~36% coverage) in the *M. bovis* strains, except for the Rv3117 locus. Interestingly, the RD5^mic^ region was largely absent from the *M. bovis* and *M. microti* OV254 genomes, but was almost completely covered in the three Belgian *M. microti* strains. In contrast to *M. microti* OV254, the *plcC* (Rv2349c), *plcB* (Rv2350c), *plcA* (Rv2351c), and PPE38 (Rv2352c) loci were completely covered in the three Belgian strains. The MiD3 region was present in all *M. bovis* strains under study, but was below the detection threshold in all *M. microti* strains. However, the *M. microti* strains contained a large fraction of the MiD3 region, but no intact ORFs for PE_PGRS50 (Rv3345c), a transmembrane protein (Rv3346c), PPE55 (Rv3347c), and a transposase (Rv3349c). Only the transposase Rv3348c had an intact ORF. The RD1^mic^, RD1^bcg^, RD3, and RD149 regions were present in *M. bovis* AF2122/97 and *M. bovis* MB3601, but were absent in all other strains (*M. bovis* BCG and *M. microti* strains). The beginning of the RD1^bcg^ region (Rv3871-Rv3879c) was missing in the *M. microti* strains, as was most of the RD1^mic^ region (Rv3864-Rv3876). For the RD3 region (prophage phiRv1; Rv1573-Rv1586c), which partially overlaps with the RD149 region (Rv1585c-Rv1587c), only the Rv1587c ORF was intact in *M. bovis* BCG and the *M. microti* strains. The RD2 region was present in all strains, except for *M. bovis* BCG Danish 1311. Lastly, the partially overlapping RD207 and MiD1 regions were present in all MTBC strains except for *M. microti* OV254, resulting in missing or interrupted CRISPR-Cas loci *cas2* (Rv2816c), *cas1* (Rv2817c), *csm6* (Rv2818c), and *csm5* (Rv2819c). Similarly, the MiD2 region (Rv3188-Rv3189) was present in all analyzed MTBC strains, except for *M. microti* OV254.

The presence of the RDs and MiDs in all *M. microti* strains used in the phylogenomic analysis (i.e., the 22 *M. microti* strains) is shown in Supplementary Figure 1. For most of the regions tested, the coverage was very similar between all *M. microti* strains. However, some variability between strains were observed for the RD5^mic^ and RD6 regions, which were almost completely covered in Belgian strains but partially missing or absent in some other strains. The MiD2 region was present in all *M. microti* strains, except the reference OV254. Similarly, the RD724 region was present in all *M. microti* strains except the strain G25822. Differences were observed for the CRISPR-Cas loci (i.e., MiD1/RD207 regions) which were fully present in ATCC 35782, ATCC 19422, 416/01, 8753/00, *microti*-45, and three Belgian strains, but (partially) absent in other strains, including the 94-2272, Maus III, Maus IV, *microti*-36, *microti*-68 and G25821-G25828 strains (i.e., the *cas2* and *cas1* genes absent and the *csm6* gene interrupted) and the OV254 reference strain. While this analysis reveals the presence of genomic regions, it does not provide information on the expression of the ORFs located in these regions, as they may be affected by SNPs and indels.

### Pan-genome analysis and identification of polymorphism signals

The pan-genome analysis detected a total of 4,668 genes, of which 1,401 were annotated as hypothetical proteins. A schematic representation of the overlap between species is shown in Supplementary Figure 2. To obtain genes that could be potential biomarkers to discriminate *M. microti* from *M. bovis*, the genes present exclusively in the two virulent *M. bovis* or in all *M. microti* (*n* = 22) strains studied were extracted (Supplementary Figure 2). Note that, for the purpose of this study, the results for the *M. bovis* BCG Danish 1311 strains were not included in this analysis, because this vaccine strain differs from the traditional virulent strains. The resulting selection is shown in Table 3 and was performed from 47 and 34 genes exclusively present in virulent *M. bovis* and *M. microti* strains, respectively. Of these 81 genes, 30 were annotated as hypothetical proteins (i.e., 19 *M. bovis* and 11 *M. microti* exclusive genes).

**Table 3:** Potential biomarkers identified from the results of the pan-genome analysis.

**A. Protein-encoding genes deleted in all** ***M. microti*** **strains**^**b**^		
**Gene_ID/Locus_tag** ^a^	**Protein_Name**	**Characteristics**
FCU26_1135	PE-PGRS family protein PE_PGRS21	Deleted
Rv3018A	PE family protein PE27A	Deleted; associated to MiD4
Rv3019c	ESAT-6-like protein EsxR	Deleted; associated to MiD4
Rv3020c	ESAT-6-like protein EsxS	Deleted; associated to MiD4
Rv3021c/Rv3022c	Uncharacterized PPE family protein PPE47/PPE48	Deleted; associated to MiD4
BQ2027_RS02780	Hypothetical protein; Rv0530A	Deleted
BQ2027_RS20600	Hypothetical protein	Deleted
BQ2027_RS17270	Transposase	Deleted
**B. Protein-encoding genes deleted in all virulent** ***M. bovis*** **strains**^c^		
**Gene/Locus_tag**	**Protein_Name**	**Characteristics**
Rv1508A	NAD(P)-binding domain-containing protein	Deleted
Rv3162c (JN986_RS16415)	Integral membrane protein	Deleted
JN986_RS12585	Hypothetical protein	Deleted
**C. Protein-encoding genes deleted in Belgian** ***M. microti*** **strains**^d^		
**Gene/Locus_tag**	**Protein_Name**	**Characteristics**
Rv0833	PE-PGRS family protein PE_PGRS13	Deleted
BQ2027_RS18035	PE family protein	Partially deleted
BQ2027_RS18040	PE family protein	Deleted
BQ2027_RS06585	F420H(2)-dependent quinone reductase	Interrupted CDS
BQ2027_RS16095	NAD(P)H nitroreductase	Deleted
BQ2027_RS16100	Hypothetical protein	Deleted
BQ2027_RS16105	Transposase	Deleted
BQ2027_RS03930	PE-PGRS family protein PE_PGRS9	Deleted
BQ2027_RS19970	Alanine and proline rich membrane-anchored mycosin mycP2	Deleted
JN986_RS07610	PE family protein	Deleted
BQ2027_RS09100	Glycosyltransferase (ORF2)	Deleted; associated to RvD2
BQ2027_RS09105	Sulfite oxidase (ORF3)	Deleted; associated to RvD2
BQ2027_RS09110	RND family transporter (ORF4)	Deleted; associated to RvD2
BQ2027_RS09115	Cutinase Cut1	Deleted; associated to RD152
BQ2027_RS09120	PE family protein Wag22a	Deleted; associated to RD152
BQ2027_RS09125	PE family protein Wag22b	Deleted; associated to RD152
BQ2027_RS09130	DUF5073 family protein	Deleted; associated to RD152
BQ2027_RS09135	Heavy metal-binding domain-containing protein	Deleted; associated to RD152
BQ2027_RS09140	Hypothetical protein	Deleted; associated to RD152
**D. Protein-encoding genes deleted only in** ***M. microti*** **OV254**^e^		
**Gene/Locus_tag**	**Protein_Name**	**Characteristics**
BQ2027_RS03980	PPE family protein	Interrupted CDS
BQ2027_RS16400	DUF2384 domain-containing protein	Deleted
BQ2027_RS16405	RES family NAD+ phosphorylase	Deleted
BQ2027_RS04800	Acetyl-CoA acetyltransferase	Interrupted CDS
BQ2027_RS07120	Hypothetical protein	Deleted

^a^Locus_tags noted “Rv,” “BQ2027,” “JN986,” and “FCU26” were associated to, respectively, *M. tuberculosis* H37Rv, *M. bovis* AF2122/97, *M. microti* OV254, and *M. bovis* BCG Danish 1311 genomes.

^b^Deleted in all *M. microti* (n=22) strains studied but present in *M. bovis* AF2122/97 and MB3601 strains.

^c^Deleted in *M. bovis* AF2122/97 and MB3601 strains but present in all *M. microti* (n=22) strains studied.

^d^Deleted in Belgian *M. microti* MI20-1, MI20-2, and VAR696 strains and other strains studied, except *M. microti* OV254 and *M. bovis* strains.

^e^Deleted in *M. microti* OV254 strain but present in the other *M. microti* and *M. bovis* strains.

bp, base pair; CDS, coding DNA sequence. Note that the locus tag corresponds to the closest matching sequence from the reference gene database and does not necessarily correspond to a gene with the same sequence and/or function.

Among the 47 genes exclusively present in virulent *M. bovis* (including ORFs located in RD1^mic^, MiD3 and RD3 regions) were several genes encoding for proline-glutamate (PE) and proline-proline-glutamate (PPE) proteins, as well as the EsxR (Rv3019c) and EsxS (Rv3020c) proteins. This deletion, corresponding to the MiD4 region (Rv3018c-Rv3022c), was previously reported in the *M. microti* OV254 strain and other *M. microti* strains isolated from patients (40). Other protein-encoding genes, such as PE_PGRS21 (FCU26_1135), the hypothetical protein Rv0530A (locus_tag BQ2027_RS02780), a transposase (BQ2027_RS17270), and the hypothetical protein (WP_015631092.1; locus_tag BQ2027_RS20600) were also absent in all 22 *M. microti* strains studied and present in the virulent *M. bovis* strains (Table 3, point A).

In addition to the genes associated with the RD4, RD11, RD12^bov^, and RD13 regions (mostly absent in *M. bovis*), three other additional genes were identified among 34 genes that were exclusively present in all *M. microti*. These genes encode the hypothetical protein Rv1508A, a hypothetical protein (JN986_RS12585) and the integral membrane protein Rv3162c. Further investigation revealed that the Rv3162c gene was present in the *M. bovis* genomes, but it contained a single nucleotide deletion resulting in an inactive gene product (Table 3, point B). Of note, some loci recorded as deleted in *M. microti* or *M. bovis* strains in the pan-genome are likely artifacts related to the Roary method used in this work, which is based on (imperfect) assembled genomes and annotations. Other genes were present in multiple copies, with different variants, and their presence or absence could not be confirmed.

Furthermore, we investigated the differences between the *M. microti* strains studied in this work by focusing on the three Belgian strains and the OV254 reference strain. Three ORFs (ORF2-ORF4) were found to be deleted or interrupted in the Belgian *M. microti* strains. These were associated with the RvD2 region including *plcD*, a glycosyltransferase (ORF2), a sulfite oxidase (ORF3), and a RND family transporter (ORF4), known to be absent in the *M. tuberculosis* H37Rv strain (H37Rv related deletion region or RvD). The region adjacent to RvD2, called RD152, was completely absent in these three strains. These two regions were also found to be partially (RvD2) or completely (RD152) absent in the *M. microti*-45 strain, the phylogenetically closest strain of the three Belgian strains. In addition, the RD152 region was found to be absent in the G25824 strain. Other genes were found to be deleted in the three Belgian strains, but present in the OV254 and the *M. bovis* strains (Table 3, point C). In addition to the genes associated with RDs/MiDs exclusively absent in *M. microti* OV254, five other genes were identified during the pan-genome analysis as shown in Table 3, point D.

### Identification of protein-encoding genes as potential diagnosis markers

Proteins encoded by the genes from RDs present in virulent *M. bovis* strains and absent in all *M. microti* strains studied (RD1^mic^, RD3, and MiD3) and vice versa (RD4, RD11, RD12^bov^, RD13, and N-RD25^bov/cap^), as well as the proteins with presence/absence variation between virulent *M. bovis* and *M. microti* strains were further analyzed. As the aim was to identify “universal” antigens, genes that exhibited presence/absence variation between the different *M. microti* strains were not considered.

Of these 80 proteins, 52 were predicted to have a signal peptide, to be non-classically secreted and/or, to be located in the extracellular matrix by the SignalP6.0, SecretomeP2.0, Psortb3.0, Gpos-mPLoc, and CELLO2.5 tools. Protein-BLAST revealed that 27 of these 52 proteins had little or no homology to any sequence of MAC members in the database. Three proteins belonging to the PE/PPE family protein (PE_PGRS50, PPE55 and PE_PGRS21) showed sequence similarity between 40 and 60% but a very low query coverage (5–18%) in the aligned sequences, and were retained in this work. The antigenic propensity of all of these 30 proteins was then evaluated using the VaxiJen 2.0 and ANTIGENpro servers.

After these selection steps, a total of 28 proteins remained that were predicted by at least one of the tools to be secreted or located on the cell surface, with low conservation in environmental mycobacteria and putative antigenic properties (Table 4 and Supplementary Tables 3–5).

**Table 4:** Selected proteins from RDs and pan-genome analysis.

**Locus_tag^a^**	**Protein_name (characterization)**	**Length (amino acid)**	***M. bovis***	***M. microti***
Rv2645	Uncharacterized protein Rv2645	143	-	+
Rv2647	Resolvase/invertase-type recombinase catalytic domain-containing protein	122	-	+
Rv2650c	Possible PhiRv2 prophage protein	479	-	+
Rv2653c	Toxin Rv2653c	107	-	+
Rv2656c	Uncharacterized protein Rv2656c	130	-	+
Rv3120	Methyltransf_25 domain-containing protein	200	-	+
Rv3738c	Uncharacterized PPE family protein PPE66	315	-	+
Rv3864	ESX-1 secretion-associated protein EspE	402	+	-
Rv3865	ESX-1 secretion-associated protein EspF	103	+	-
Rv3866	ESX-1 secretion-associated protein EspG1	283	+	-
Rv3867	ESX-1 secretion-associated protein EspH	183	+	-
Rv3869	ESX-1 secretion system ATPase EccB1	480	+	-
Rv3872	PE family immunomodulator PE35	99	+	-
Rv3873	PPE family immunomodulator PPE68	368	+	-
Rv3874	ESAT-6-like protein EsxB (CFP10)	100	+	-
Rv3875	6 kDa early secretory antigenic target (ESAT-6)	95	+	-
Rv3876	ESX-1 secretion-associated protein EspI	666	+	-
Rv1573	Probable PhiRv1 phage protein	136	+	-
Rv1574	Probable PhiRv1 phage related protein	103	+	-
Rv1575	Probable PhiRv1 phage protein	166	+	-
Rv1578c	Probable PhiRv1 phage protein	156	+	-
Rv1579c	Probable PhiRv1 phage protein	104	+	-
Rv1580c	Probable PhiRv1 phage protein	90	+	-
Rv1581c	Probable PhiRv1 phage protein	131	+	-
Rv3345c	PE-PGRS family protein PE_PGRS50	1,538	+	-
Rv3347c	PPE family protein PPE55	3,157	+	-
BQ2027_RS20600	Hypothetical protein	139	+	-
FCU26_1135	PE-PGRS family protein PE_PGRS21	809	+	-

Proteins, predicted as extracellular, antigenic and as having a low sequence similarity with environmental mycobacteria, were selected.

^a^Locus_tags noted “Rv,” “BQ2027,” and “FCU26” were associated to, respectively, *M. tuberculosis* H37Rv, *M. bovis* AF2122/97, and *M. bovis* Danish 1311 genomes. +, present; - absent in two virulent *M. bovis* or in all *M. microti* (n = 22) strains studied.

### Prediction of sero-dominant epitopes

A total of 157 regions predicted as both linear and conformational epitopes with an antigenicity score >0.5 in the VaxiJen 2.0 server were identified and considered as promising epitopes for serodiagnosis. The peptide sequences with the highest antigenicity score are shown in Table 5 for the 28 proteins analyzed.

**Table 5:** Final list of the B-cell epitopes from the *in silico* analysis.

**Locus_tag^a^**	**No. linear epitopes predicted**	**No. discontinuous epitopes predicted**	**No. final epitopes predicted**	**Selected B-cell epitope**	**No. residues**	**Start-stop positions**	**Score Vaxijen2.0**
Rv2645	6	13	3	LFCAHADTNGDPGRCACGQQLADVG	25	9–33	1.0323
Rv2647	4	5	3	QRQREGGDHGRQARYSGLGSMEPKP	25	49–73	1.5047
Rv2650c	27	21	12	GTADYTVTGAGTEAVVAADV	20	324–343	1.4269
Rv2653c	5	10	4	AGMKLGWHPYHFPDEPDSKQ	20	88–107	1.0662
Rv2656c	6	6	3	MTAVGGSPPTRRCPATE	17	1–17	1.0279
Rv3120	9	15	5	VQLELEATRRELADR	15	83–97	0.9598
Rv3738c	17	14	6	AIEVGLKTADVAPVAVRPAAAPP	23	207–229	0.8783
Rv3864	21	27	5	QAQQGGQQHATLVSDK	16	356–371	1.2968
Rv3865	5	5	2	KADDGLAGVI	10	89–98	1.0464
Rv3866	16	15	6	RHPGGTTTQTD	11	207–217	1.7303
Rv3867	9	12	4	SPLDALDDYAPVQTDDAEGD	20	28–47	0.975
Rv3869	21	28	7	LQSPDPRYTES	11	397–407	1.5677
Rv3872	6	5	2	KMSHDPIAAD	10	3–12	0.9431
Rv3873	18	21	9	TGGAAPVGAGAMGQGAQSGGSTR	23	319–341	1.8743
Rv3874	4	9	3	EISTNIRQAGVQYSRADEEQ	20	71–90	1.0216
Rv3875	6	10	3	AAAWGGSGSEAYQGVQQ	17	40–56	0.9832
Rv3876	36	38	12	GPSIQARLRAEEASGA	16	246–261	1.6683
Rv1573	8	8	5	ADAAGGDECGPAI	13	76–88	1.9176
Rv1574	4	8	4	MGYKPESERH	10	1–10	1.3071
Rv1575	8	11	4	EPKPSQRHTDKEV	13	2–14	1.2911
Rv1578	10	10	3	GDDGDGLNPFAPD	13	143–155	1.1461
Rv1579	7	16	2	ARPIEQDDDQGAGSPS	16	89–104	1.3097
Rv1580	5	9	4	PYIILPNLQTGEPVMGVA	18	36–53	1.1495
Rv1581	7	10	1	VAITPASGGRHSVRF	15	4–18	1.2328
Rv3345c	49	49	11	GAAGGNGGAGAGGGSLSTGQSGGPR	25	1,430–1,454	2.6549
Rv3347c	87	25	12	SSSGFKVRPSFSFFAV	16	2,928–2,943	1.6803
BQ2027_RS20600	5	8	3	GGGGGGGGADTNRSD	15	73–87	4.1956
FCU26_1135/PE_PGRS21	34	41	19	GTGGLGNRGGAGG	13	292–305	2.8749
**Total**	440	449	157	-	-	-	-

^a^Locus_tags noted “Rv,” “BQ2027,” and “FCU26” were associated to, respectively, *M. tuberculosis* H37Rv, *M. bovis* AF2122/97, and *M. bovis* BCG Danish 1311 genomes.

The Vaxijen2.0 threshold was set to 0.5.

Only B-cell epitopes with the highest Vaxijen2.0 score are shown.

## Discussion

*M. microti* is less virulent in humans and livestock than other members of the MTBC (51), and is not considered as a causative agent of TB in animals under the new animal health law (EU 2016/429). However, its spread and the increasing number of reported cases of infection in different hosts (9–11, 13, 76, 77) is a concern for the identification and management of control strategies for TB in animals, especially when the prevalence of animal TB is low. Field data suggest that *M. microti* may interfere with various *ante-mortem* diagnostic tests used in animal TB surveillance programs in livestock, camelids and other species [unpublished data from the Belgian NRL; (11)].

In this study, we first performed WGS on three *M. microti* field strains and compared the genomic diversity within the publicly available *M. microti* WGS data. Secondly, we compared the complete genomes of different *M. microti* and *M. bovis* strains, through characterization of large sequence polymorphisms (RDs, MiDs, and RvD) and pan-genomic analysis to identify potential markers for discriminating between the two mycobacteria in animal TB diagnosis. The analysis focused on proteins that are likely to be suitable for serological diagnostic tests. Serological assays are the primary *ante-mortem* tests used to detect TB in camelids [due to the poor performance of the IFNɤ and SICCT assays in these hosts; (4)] but, there are currently no assays available to distinguish between *M. microti* and *M. bovis* infections. An *in silico* approach was then used to select the best B-cell epitopes from these proteins for use in serodiagnosis.

The phylogenomic analysis provided insight into the genetic diversity within the *M. microti* species, including differences between isolates collected from different host species. As previously shown by (39), *M. microti* strains tended to cluster based on their spoligopattern rather than their host species. However, the bootstrap support for the relationships between clades was very low, indicating low confidence in the relative placement of the clades, possibly due to the limited WGS data available for *M. microti*. We chose to harmonize the spoligotype detection by determining it *in silico* using the WGS datasets to reduce bias between samples. In some cases, the spoligotypes that were extracted from the WGS data differed slightly from those previously reported (20, 38), which may be due to the inherent difficulty in determining spoligotypes, both in dry-lab and wet-lab techniques. SNP distances between *M. microti* strains isolated from Belgian alpaca farms could provide an indication of potential epidemiological links between strains. The mean substitution rate of *M. tuberculosis* has been estimated to be 0.3–0.5 SNPs per genome per year and SNP thresholds ranging from 5 (stringent, most likely) to 12 SNPs (more putative) have been defined to infer transmission events (78, 79). Several studies carried out on *M. bovis* isolates suggest that a similar rate might be applicable for animal-adapted mycobacterial species (78, 80–83). Based on these SNP thresholds, *M. microti* strains VAR696 and MI20-1, MI20-2, isolated from two different Belgian farms and differing by 18 SNPs, were unlikely to be epidemiologically linked. In contrast, the two *M. microti* strains MI20-1 and MI20-2 isolated from the same farm and showing 8 SNPs differences, could be related to a past transmission event or past epidemiological interactions within the same farm. However, these SNP profiles could also be caused by different past introductions, by the introduction of infected animals (with different genotypes) from different locations, or by different strains circulating on the same farm or in the region.

Genomic variations, such as RD or SNP, are markers used to study the evolution or epidemiological relationship existing between the species of the MTBC or the strains of the same species (82, 84). These variations may also be useful as diagnostic markers. In this study, the genomic differences between the tested *M. microti* strains were first investigated by comparing the RDs and MiDs. Little variation was observed between the *M. microti* strains for most of the tested regions. As previously reported, the *M. microti* strains are all characterized by the deletion of the RD1^mic^ region, which contains several genes encoding the ESX-1 secretion system, including the virulence factors ESAT6 (Rv3875) and CFP10 (Rv3874). The MiD3, MiD4, and RD3 regions were also absent in all tested *M. microti* strains, including the Belgian *M. microti* strains (20, 39, 40). However, differences were observed between the different *M. microti* strains for some RDs/MiD, such as RD5^mic^, RD6, RD207/MiD1, and MiD2. The RD5 region contains the phospholipase C loci (*plcA, plcB*, and *plcC*), and is highly variable between different species of tuberculous mycobacteria but also between strains of the same species as shown by (21) and (39). The RD6 region, containing the IS*1532* element, is also described as highly variable among MTBC strains (21). The presence or (partial) deletion of the RD207 region (which includes the MiD1 region) in the *M. microti* strains results in the loss of spacers in the DR locus and would therefore be associated with the llama and vole spoligotypes observed (20, 85). Since screening for RDs is limited to regions of the H37Rv *M. tuberculosis* genome, a pan-genomic analysis was performed in order to reveal additional differences. This analysis revealed the absence of several genes which, after more detailed investigation, were identified to be associated with RvD2 and RD152 regions. These two regions were only found (partially) deleted in the Belgian strains and *M. microti*-45, but were fully present in the reference OV254 and other *M. microti* strains studied (except for the RD152 region found to be also deleted in the G25824 strain). The RvD2 region is located downstream of the *plcD* gene (Rv1755c) and encodes three ORFs (Mb1785-Mb1787) predicted to be a hypothetical protein, a putative sulfite oxidase and a putative transmembrane transport protein (86–88). This region is known to be absent in *M. tuberculosis* H37Rv but is present in other members of the MTBC such as *M. bovis*. The RD152 region encodes genes Rv1758-Rv1765c (89), including a putative cutinase (cut1) and a member of the PE_PGRS protein family (wag22). Deletions of the RD152 region have previously been identified in *M. africanum* lineage nine strains (90) and *M. tuberculosis* Beijing/W strains (91). To our knowledge, these deletions have never been reported in *M. microti* or other animal-adapted MTBC species. Both regions have been reported as hotspots for IS*6110* insertion sequences, which may facilitate recombination events (20, 85, 92). Thus, an IS*6110*-mediated deletion mechanism could be responsible for the loss of the RvD2 and RD152 regions in these strains. However, strains with these deletions were still capable of causing tuberculosis lesions in alpacas, as observed with Belgian *M. microti* strains, suggesting that these regions may not be essential for virulence.

Genes showing presence-absence variation were selected as potential protein markers to distinguish *M. bovis* from *M. microti* infections. A total of 80 protein-encoding genes were identified that were exclusively present in all *M. microti* strains tested and absent in all virulent *M. bovis* strains tested, or *vice versa*. The gene detection method employed in the pan-genome analysis was, in some cases, based on fragmented genome assemblies. Therefore, genes may have been missed due to assembly fractures, or other inaccuracies in the assembled genomes or gene predictions, highlighting the need for *in vitro* verification. Nevertheless, these types of analyses are a powerful and rapid screening method that can be used to generate peptide libraries that can be tested *in vitro*. Many potential biomarkers identified in the present pan-genome analysis have previously been reported as potential antigens to improve the serological diagnosis of human and/or bovine tuberculosis: Rv3872 (PE35), Rv3874 (CFP10), Rv3875 (ESAT6), Rv3876 (EspI) in RD1 (93–96), Rv1573, Rv1577c in RD3 (97), or Rv1508c, Rv1509, Rv1514c, Rv1516c in RD4 (93, 97). In addition, several of the genes with presence-absence variation identified in this study encode proteins belonging to the PE, PPE, and Polymorphic GC-Rich Sequence (PE_PGRS) families, including PE_PGRS21, PPE55, and PE_PGRS50. The proteins of these families, which constitute ~10% of the *M. bovis* genome (22), have been described as virulence factors involved in the pathogenesis of tuberculosis, but also as having a potential role in the antigenic variability within the MTBC (98, 99). In addition, several proteins of the PE/PPE/PE_PGRS family have been reported to be immunogenic and may be useful in the diagnosis of human and/or animal tuberculosis (98, 100). It is noteworthy that the antigens ESAT6 and CFP10, which are used in various assays for diagnosis of TB in animals (e.g., the IFNɤ assay and the Enferplex Camelid TB test), are absent in the *M. microti* strains but present in the *M. bovis* strains (7, 101, 102). Consequently, the absence of an antibody response to these antigens does not confirm the absence of *M. microti* infection, highlighting the importance of *M. microti*-specific antigens.

The main problem in establishing immunodiagnostic systems is the identification of immunogenic proteins specific to the target species. Several additional *in silico* analyses were performed to obtain a set of optimal potential immunogen proteins, including an evaluation of sequence homology, antigenic probability and accessibility by antibodies. Proteins with high sequence similarity/identity to environmental mycobacteria, such as members of the MAC commonly isolated from cattle, may cross-react in serological tests, and were therefore excluded (61, 63). After this additional filtering, a total of 28 candidate proteins were retained. However, it is possible that effective antigens were removed by this filtering. For example, proteins such as Rv3871 (103), Rv1509, Rv2658c (97), and Rv1586c (104) were not predicted to be secreted/located on the cell surface and/or antigenic in this work, whereas they have been reported to induce an antibody response in TB patients. As our aim was to maximize the detection of *M. bovis* vs. *M. microti* infection cases, only “universal” protein antigens present in all *M. microti* strains under study but absent in all virulent *M. bovis* strains (and *vice-versa*) were considered. Although genes that were absent/present in the Belgian *M. microti* strains (and some other strains) were not considered as potential antigens in this work, further investigation of these proteins may be valuable.

The production of recombinant proteins to obtain mycobacterial antigens is a challenging task associated with high costs and antigen variability related to expression systems and purification steps for recombinant proteins. The use of synthetic peptides can provide uniformity in antigen preparations and thus in assays (105). Therefore, the identification of peptides containing immunodominant B-cell epitopes may be essential for the development of diagnostic strategies aimed at antibody detection. An additional advantage is the reduced cost associated with the number of peptides for which antibody reactivity must be evaluated experimentally. Many studies have focused primarily on linear B-cell epitopes, whereas an important fraction of the B-cell epitopes are conformational (106). Until recently, the prediction of conformational B-cell epitopes required the protein structure to be deposited in the PDB database or the use of homology modeling tools (106, 107). However, the recently released neural network-based AlphaFold is a powerful alternative that enables highly accurate protein structure prediction (74). In this work, peptides based on both linear and conformational B-cell epitopes were sought using various *in silico* tools available online to predict B-cell epitopes based on amino acid sequences and/or protein structural data (74, 108). Therefore, a total of 157 peptides containing predicted B-cell epitopes were identified from the 28 previously selected proteins using the *in silico* approach. Interestingly, some peptides, which were derived from the full sequence of the candidate proteins, such as the peptide EISTNIRQAGVQYSRADEEQ for RV3874 (109) or the peptide AGMKLGWHPYHFPDEPDSKQ for Rv2653c (110), were previously reported to induce a moderate to strong CMI response in tuberculous animals or patients, suggesting that these peptides could potentially activate both humoral and cellular immunity and have a potential in various diagnostic applications. However, no information was found in the scientific literature on the recognition of these specific peptides by antibodies in a serological assay.

*In silico* assays can guide the experimental testing to screen for putative antigens more efficiently. In this paper, an *in silico* approach was used to identify putative antigenic peptides containing B-cell epitopes that discriminate between *M. bovis* and *M. microti* infections in animal TB serodiagnosis. *In vitro* analyses are nevertheless required to identify immunologically relevant peptides among the ones selected *in silico*. The immunoreactivity of the peptides should be evaluated by immunoassay (e.g., ELISA) with a sufficient number of reference sera, i.e. sera from presumed uninfected animals with a known status and sera from animals with *M. bovis* or *M. microti* infection confirmed by bacterial culture and molecular methods. The peptides with the highest sensitivity and specificity values will be further assessed for their serodiagnostic ability in a larger panel of sera. Previous data reported that the antibody response to antigens can be boosted in cattle, alpacas, goats following the intradermal tuberculin test, which can impact the performance of serological tests, in particular sensitivity (4, 14, 109). This should be considered in the future studies. Furthermore, although the use of carefully selected synthetic peptides decrease the inclusion of cross-reactive region of the protein antigens, it might be necessary to confirm the absence of aspecific response in the test using sera from NTM-infected animals. As combinations of mycobacterial antigens (peptides or recombinant proteins) are indicated to improve serodiagnosis of tuberculosis in humans and animals (16, 105, 111), the diagnostic performance of peptides confirmed as immunogenic in preliminary assays could be further evaluated in multiplexed serological assays or by polypeptide antigens.

Using WGS, we were able to extract potential biomarkers across the entire genome. In addition, whole-genome SNP-based analysis allowed us to characterize relationships between strains. As the number of *M. microti* WGS datasets increases, to which our study contributes, the effectiveness of these WGS-based analyses may increase further. In conclusion, the results presented in this study may contribute to the development of serological tests that can differentiate between *M. bovis* and *M. microti* in the future, thus avoiding misdiagnosis in the control TB in animals.

## Data Availability

The datasets presented in this study can be found in online repositories. The names of the repository/repositories and accession number(s) can be found in the article/Supplementary material.

## Supplementary material

Supplementary Figure 1 Presence of RDs and MiDs regions in the *M. microti* datasets. This figure shows the percentage of the corresponding RDs and MiDs regions that were covered by the read mapping-based approach for each dataset.

Supplementary Figure 2 Results of the pan-genome analysis, showing the number of genes detected exclusively in all *M. microti* and all virulent *M. bovis* strains. Note that all 22 *M. microti* strains and the two virulent *M. bovis* strains (AF2122/97 and MB3601) were considered. The horizontal bars indicate the total number of genes detected in the corresponding species by the pan-genome analysis. The vertical lines indicate the size of the overlap as indicated by the dots in membership matrix.

## References

[1] WOAH. Infection with *Mycobacterium tuberculosis* complex. In: World Organisation for Animal Health—Terrestrial Animal Health Code, Chapter 8.12. Paris (2023).

[2] Palmer MV Thacker TC Waters WR Gortázar C Corner LAL. *Mycobacterium bovis*: a model pathogen at the interface of livestock, wildlife, and humans. Vet Med Int. (2012) 2012:e236205. 10.1155/2012/236205 22737588 PMC3377356

[3] García-Bocanegra I Barranco I Rodríguez-Gómez IM Pérez B Gómez-Laguna J Rodríguez S. Tuberculosis in alpacas (*Lama pacos*) caused by *Mycobacterium bovis*. J Clin Microbiol. (2010) 48:1960–4. 10.1128/JCM.02518-09 20237097 PMC2863934

[4] Rhodes S Holder T Clifford D Dexter I Brewer J Smith N. Evaluation of gamma interferon and antibody tuberculosis tests in alpacas. Clin Vac Immunol. (2012) 19:1677–83. 10.1128/CVI.00405-12 22914362 PMC3485894

[5] Lyashchenko KP Greenwald R Esfandiari J Meylan M Burri IH Zanolari P. Antibody responses in New World camelids with tuberculosis caused by *Mycobacterium microti*. Vet Microbiol. (2007) 125:265–73. 10.1016/j.vetmic.2007.05.026 17628360

[6] Bezos J Casal C Álvarez J Díez-Guerrier A Rodríguez-Bertos A Romero B. Evaluation of the performance of cellular and serological diagnostic tests for the diagnosis of tuberculosis in an alpaca (*Vicugna pacos*) herd naturally infected with *Mycobacterium bovis*. Prev Vet Med. (2013) 111:304–13. 10.1016/j.prevetmed.2013.05.013 23809774

[7] Krajewska-Wedzina M Didkowska A Sridhara AA Elahi R Johnathan-Lee A Radulski Ł. Transboundary tuberculosis: importation of alpacas infected with *Mycobacterium bovis* from the United Kingdom to Poland and potential for serodiagnostic assays in detecting tuberculin skin test false-negative animals. Transbound Emerg Dis. (2020) 67:1306–14. 10.1111/tbed.13471 31899584

[8] Kasai H Ezaki T Harayama S. Differentiation of phylogenetically related slowly growing mycobacteria by their gyrB sequences. J Clin Microbiol. (2000) 38:301–8. 10.1128/JCM.38.1.301-308.2000 10618105 PMC88713

[9] Ghielmetti G Kupca AM Hanczaruk M Friedel U Weinberger H Revilla-Fernández S. *Mycobacterium microti* infections in free-ranging red deer (*Cervus elaphus*). Emerg Infect Dis. (2021) 27:2025–32. 10.3201/eid2708.210634 34286688 PMC8314804

[10] Michelet L de Cruz K Zanella G Aaziz R Bulach T Karoui C. Infection with *Mycobacterium microti* in animals in France. J Clin Microbiol. (2015) 53:981–5. 10.1128/JCM.02713-14 25540404 PMC4390619

[11] Michelet L de Cruz K Tambosco J Hénault S Boschiroli M-L. *Mycobacterium microti* interferes with bovine tuberculosis surveillance. Microorganisms. (2020) 8:1850. 10.3390/microorganisms8121850 33255311 PMC7761213

[12] Smith NH Crawshaw T Parry J Birtles RJ. *Mycobacterium microti*: more diverse than previously thought. J Clin Microbiol. (2009) 47:2551–9. 10.1128/JCM.00638-09 19535520 PMC2725668

[13] Tagliapietra V Boniotti MB Mangeli A Karaman I Alborali G Chiari M. *Mycobacterium microti* at the environment and wildlife interface. Microorganisms. (2021) 9:2084. 10.3390/microorganisms9102084 34683407 PMC8539169

[14] Infantes-Lorenzo JA Whitehead CE Moreno I Bezos J Roy A Dominguez L. Development and evaluation of a serological assay for the diagnosis of tuberculosis in alpacas and llamas. Front Vet Sci. (2018) 5:189. 10.3389/fvets.2018.00189 30151368 PMC6099158

[15] Infantes-Lorenzo JA Dave D Moreno I Anderson P Lesellier S Gormley E. New serological platform for detecting antibodies against *Mycobacterium tuberculosis* complex in European badgers. Vet Med Sci. (2019) 5:61–9. 10.1002/vms3.134 30656864 PMC6376137

[16] O'Brien A Clarke J Hayton A Adler A Cutler K Shaw DJ. Diagnostic accuracy of the Enferplex Bovine Tuberculosis antibody test in cattle sera. Sci Rep. (2023) 13:1875. 10.1038/s41598-023-28410-9 36726018 PMC9892036

[17] Lo Y-T Shih T-C Pai T-W Ho L-P Wu J-L Chou H-Y. Conformational epitope matching and prediction based on protein surface spiral features. BMC Genom. (2021) 22:116. 10.1186/s12864-020-07303-5 34058977 PMC8165135

[18] Aranaz A Liébana E Mateos A Dominguez L Vidal D Domingo M. Spacer oligonucleotide typing of *Mycobacterium bovis* strains from cattle and other animals: a tool for studying epidemiology of tuberculosis. J Clin Microbiol. (1996) 34:2734–40. 10.1128/jcm.34.11.2734-2740.1996 8897175 PMC229396

[19] Kremer K van Soolingen D van Embden J Hughes S Inwald J Hewinson G. *Mycobacterium microti*: more widespread than previously thought. J Clin Microbiol. (1998) 36:2793–4. 10.1128/JCM.36.9.2793-2794.1998 9742014 PMC105214

[20] Brodin P Eiglmeier K Marmiesse M Billault A Garnier T Niemann S. Bacterial artificial chromosome-based comparative genomic analysis identifies *Mycobacterium microti* as a natural ESAT-6 deletion mutant. Infect Immun. (2002) 70:5568–78. 10.1128/IAI.70.10.5568-5578.2002 12228284 PMC128332

[21] Brosch R Gordon SV Marmiesse M Brodin P Buchrieser C Eiglmeier K. A new evolutionary scenario for the *Mycobacterium tuberculosis* complex. Proc Natl Acad Sci USA. (2002) 99:3684–9. 10.1073/pnas.052548299 11891304 PMC122584

[22] Guimaraes AMS Zimpel CK. *Mycobacterium bovis*: from genotyping to genome sequencing. Microorganisms. (2020) 8:667. 10.3390/microorganisms8050667 32375210 PMC7285088

[23] Landolt P Stephan R Scherrer S. Development of a new High Resolution Melting (HRM) assay for identification and differentiation of *Mycobacterium tuberculosis* complex samples. Sci Rep. (2019) 9:1850. 10.1038/s41598-018-38243-6 30755639 PMC6372708

[24] De la Fuente J Diez-Delgado I Contreras M Vicente J Cabezas-Cruz A Tobes R. Comparative genomics of field isolates of *Mycobacterium bovis* and *M. caprae* provides evidence for possible correlates with bacterial viability and virulence. PLoS Negl Trop Dis. (2015) 9:e0004232. 10.1371/journal.pntd.0004232 26583774 PMC4652870

[25] Meehan CJ Goig GA Kohl TA Verboven L Dippenaar A Ezewudo M. Whole genome sequencing of *Mycobacterium tuberculosis*: current standards and open issues. Nat Rev Microbiol. (2019) 17:533–45. 10.1038/s41579-019-0214-5 31209399

[26] Reis AC Cunha MV. Genome-wide estimation of recombination, mutation and positive selection enlightens diversification drivers of *Mycobacterium bovis*. Sci Rep. (2021) 11:18789. 10.1038/s41598-021-98226-y 34552144 PMC8458382

[27] Reis AC Cunha MV. The open pan-genome architecture and virulence landscape of *Mycobacterium bovis*. Microb Genom. (2021) 7:000664. 10.1099/mgen.0.000664 34714230 PMC8627212

[28] Lorente-Leal V Liandris E Pacciarini M Botelho A Kenny K Loyo B. Direct PCR on tissue samples to detect *Mycobacterium tuberculosis* complex: an alternative to the bacteriological culture. J Clin Microbiol. (2021) 59:20. 10.1128/JCM.01404-20 33239374 PMC8111149

[29] Thierry D Cave MD Eisenach KD Crawford JT Bates JH Gicquel B. IS6110, an IS-like element of *Mycobacterium tuberculosis* complex. Nucl Acids Res. (1990) 18:188. 10.1093/nar/18.1.188 2155396 PMC330226

[30] Kamerbeek J Schouls L Kolk A Van Agterveld M van Soolingen D Kuijper S. Simultaneous detection and strain differentiation of *Mycobacterium tuberculosis* for diagnosis and epidemiology. J Clin Microbiol. (1997) 35:907–14. 10.1128/jcm.35.4.907-914.1997 9157152 PMC229700

[31] Bogaerts B Delcourt T Soetaert K Boarbi S Ceyssens PJ Winand R. A bioinformatics whole-genome sequencing workflow for clinical *Mycobacterium tuberculosis* complex isolate analysis, validated using a reference collection extensively characterized with conventional methods and *in silico* approaches. J Clin Microbiol. (2021) 59:21. 10.1128/JCM.00202-21 33789960 PMC8316078

[32] Bolger AM Lohse M Usadel B. Trimmomatic: a flexible trimmer for illumina sequence data. Bioinformatics. (2014) 30:2114–20. 10.1093/bioinformatics/btu170 24695404 PMC4103590

[33] Prjibelski A Antipov D Meleshko D Lapidus A Korobeynikov A. Using SPAdes *de novo* assembler. Curr Protocol Bioinformat. (2020) 70:e102. 10.1002/cpbi.102 32559359

[34] Gurevich A Saveliev V Vyahhi N Tesler G. QUAST: quality assessment tool for genome assemblies. Bioinformatics. (2013) 29:1072–5. 10.1093/bioinformatics/btt086 23422339 PMC3624806

[35] Wood DE Lu J Langmead B. Improved metagenomic analysis with Kraken 2. Genome Biol. (2019) 20:257. 10.1186/s13059-019-1891-0 31779668 PMC6883579

[36] Langmead B Salzberg SL. Fast gapped-read alignment with Bowtie 2. Nat Methods. (2012) 9:357–9. 10.1038/nmeth.1923 22388286 PMC3322381

[37] Li H Handsaker B Wysoker A Fennell T Ruan J Homer N. The sequence alignment/map format and SAMtools. Bioinformatics. (2009) 25:2078–9. 10.1093/bioinformatics/btp352 19505943 PMC2723002

[38] van Soolingen D van der Zanden AGM de Haas PEW Noordhoek GT Kiers A Foudraine NA. Diagnosis of *Mycobacterium microti* infections among humans by using novel genetic markers. J Clin Microbiol. (1998) 36:1840–5. 10.1128/JCM.36.7.1840-1845.1998 9650922 PMC104938

[39] Orgeur M Frigui W Pawlik A Clark S Williams A Ates LS. Pathogenomic analyses of *Mycobacterium microti*, an ESX-1- deleted member of the *Mycobacterium tuberculosis* complex causing disease in various hosts. Microbial Genom. (2021) 7:e000505. 10.1099/mgen.0.000505 33529148 PMC8208694

[40] Garcia-Pelayo MC Caimi KC Inwald JK Hinds J Bigi F Romano MI. Microarray analysis of *Mycobacterium microti* reveals deletion of genes encoding PE-PPE proteins and ESAT-6 family antigens. Tubercul Microarrays Mycobacterium tuberculosis. (2004) 84:159–66. 10.1016/j.tube.2003.12.002 15207485

[41] Malm S Linguissi LSG Tekwu EM Vouvoungui JC Kohl TA Beckert P. New *Mycobacterium tuberculosis* complex sublineage, Brazzaville, Congo. Emerg Infect Dis. (2017) 23:423–9. 10.3201/eid2303.160679 28221129 PMC5382753

[42] Boniotti MB Gaffuri A Gelmetti D Tagliabue S Chiari M Mangeli A. Detection and molecular characterization of *Mycobacterium microti* isolates in wild boar from Northern Italy. J Clin Microbiol. (2014) 52:2834–43. 10.1128/JCM.00440-14 24871212 PMC4136184

[43] Brites D Loiseau C Menardo F Borrell S Boniotti MB Warren R. A new phylogenetic framework for the animal-adapted *Mycobacterium tuberculosis* complex. Front Microbiol. (2018) 9:2820. 10.3389/fmicb.2018.02820 30538680 PMC6277475

[44] Bogaerts B Van den Bossche A Verhaegen B Delbrassinne L Mattheus W Nouws S. Closing the gap: Oxford Nanopore Technologies R10 sequencing allows comparable results to illumina sequencing for SNP-based outbreak investigation of bacterial pathogens. J Clin Microbiol. (2024) 62:e01576–23. 10.1128/jcm.01576-23 38441926 PMC11077942

[45] Danecek P Bonfield JK Liddle J Marshall J Ohan V Pollard MO. Twelve years of SAMtools and BCFtools. GigaScience. (2021) 10:giab008. 10.1093/gigascience/giab008 33590861 PMC7931819

[46] Croucher NJ Page AJ Connor TR Delaney AJ Keane JA Bentley SD. Rapid phylogenetic analysis of large samples of recombinant bacterial whole genome sequences using Gubbins. Nucleic Acids Res. (2015) 43:e15. 10.1093/nar/gku1196 25414349 PMC4330336

[47] Arndt D Grant JR Marcu A Sajed T Pon A Liang Y. PHASTER: a better, faster version of the PHAST phage search tool. Nucleic Acids Res. (2016) 44:W16–21. 10.1093/nar/gkw387 27141966 PMC4987931

[48] Kumar S Stecher G Li M Knyaz C Tamura K. MEGA X: molecular evolutionary genetics analysis across computing platforms. Mol Biol Evol. (2018) 35:1547–9. 10.1093/molbev/msy096 29722887 PMC5967553

[49] Xia E Teo Y-Y Ong RT-H. SpoTyping: fast and accurate *in silico Mycobacterium spoligotyping* from sequence reads. Genome Med. (2016) 8:19. 10.1186/s13073-016-0270-7 26883915 PMC4756441

[50] Cole ST. Comparative and functional genomics of the *Mycobacterium tuberculosis* complex. Microbiology. (2002) 148:2919–28. 10.1099/00221287-148-10-2919 12368425

[51] Frota CC Hunt DM Buxton RS Rickman L Hinds J Kremer K. Genome structure in the vole bacillus, *Mycobacterium microti*, a member of the *Mycobacterium tuberculosis* complex with a low virulence for humans. Microbiology. (2004) 150:1519–27. 10.1099/mic.0.26660-0 15133113 PMC2964484

[52] Gagneux S DeRiemer K Van T Kato-Maeda M de Jong BC Narayanan S. Variable host-pathogen compatibility in *Mycobacterium tuberculosis*. Proc Natl Acad Sci USA. (2006) 103:2869–73. 10.1073/pnas.0511240103 16477032 PMC1413851

[53] Behr MA Wilson MA Gill WP Salamon H Schoolnik GK Rane S. Comparative genomics of BCG vaccines by whole-genome DNA microarray. Science. (1999) 284:1520–3. 10.1126/science.284.5419.1520 10348738

[54] Seemann T. Prokka: rapid prokaryotic genome annotation. Bioinformatics. (2014) 30:2068–9. 10.1093/bioinformatics/btu153 24642063

[55] Page AJ Cummins CA Hunt M Wong VK Reuter S Holden MTG. Roary: rapid large-scale prokaryote pan genome analysis. Bioinformatics. (2015) 31:3691–3. 10.1093/bioinformatics/btv421 26198102 PMC4817141

[56] Teufel F Almagro Armenteros JJ Johansen AR Gíslason MH Pihl SI Tsirigos KD. SignalP 6.0 predicts all five types of signal peptides using protein language models. Nat Biotechnol. (2022) 40:1023–5. 10.1038/s41587-021-01156-3 34980915 PMC9287161

[57] Bendtsen JD Kiemer L Fausbøll A Brunak S. Non-classical protein secretion in bacteria. BMC Microbiol. (2005) 5:58. 10.1186/1471-2180-5-58 16212653 PMC1266369

[58] Yu NY Wagner JR Laird MR Melli G Rey S Lo R. PSORTb 3.0: improved protein subcellular localization prediction with refined localization subcategories and predictive capabilities for all prokaryotes. Bioinformatics. (2010) 26:1608–15. 10.1093/bioinformatics/btq249 20472543 PMC2887053

[59] Shen H-B Chou K-C. Gpos-mPLoc: a top-down approach to improve the quality of predicting subcellular localization of gram-positive bacterial proteins. PPL. (2009) 16:1478–84. 10.2174/092986609789839322 20001911

[60] Yu C-S Chen Y-C Lu C-H Hwang J-K. Prediction of protein subcellular localization. Proteins. (2006) 64:643–51. 10.1002/prot.21018 16752418

[61] Biet F Boschiroli ML. Non-tuberculous mycobacterial infections of veterinary relevance. Res Vet Sci. (2014) 97:S69–77. 10.1016/j.rvsc.2014.08.007 25256964

[62] Infantes-Lorenzo JA Moreno I Risalde MÁ Roy Á Villar M Romero B. Proteomic characterisation of bovine and avian purified protein derivatives and identification of specific antigens for serodiagnosis of *Bovine tuberculosis*. Clin Proteom. (2017) 14:36. 10.1186/s12014-017-9171-z 29142508 PMC5669029

[63] Varela-Castro L Barral M Arnal MC Fernández de Luco D Gortázar C Garrido JM. Beyond tuberculosis: diversity and implications of non-tuberculous mycobacteria at the wildlife-livestock interface. Transbound Emerg Dis. (2022) 69:e2978-93. 10.1111/tbed.14649 35780316

[64] Magnan CN Zeller M Kayala MA Vigil A Randall A Felgner PL. High-throughput prediction of protein antigenicity using protein microarray data. Bioinformatics. (2010) 26:2936–43. 10.1093/bioinformatics/btq551 20934990 PMC2982151

[65] Doytchinova IA Flower DR. VaxiJen: a server for prediction of protective antigens, tumour antigens and subunit vaccines. BMC Bioinform. (2007) 8:4. 10.1186/1471-2105-8-4 17207271 PMC1780059

[66] Chou PY Fasman GD. Prediction of the secondary structure of proteins from their amino acid sequence. Adv Enzymol Relat Areas Mol Biol. (1978) 47:145–8. 10.1002/9780470122921.ch2 364941

[67] Emini EA Hughes JV Perlow DS Boger J. Induction of hepatitis A virus-neutralizing antibody by a virus-specific synthetic peptide. J Virol. (1985) 55:836–9. 10.1128/jvi.55.3.836-839.1985 2991600 PMC255070

[68] Karplus PA Schulz GE. Prediction of chain flexibility in proteins. Naturwissenschaften. (1985) 72:212–3. 10.1007/BF01195768

[69] Parker JMR Guo D. New hydrophilicity scale derived from high-performance liquid chromatography peptide retention data: correlation of predicted surface residues with antigenicity and X-ray-derived accessible sites? Biochemistry. (1986) 25:5425–32. 10.1021/bi00367a013 2430611

[70] Kolaskar AS Tongaonkar PC. A semi-empirical method for prediction of antigenic determinants on protein antigens. FEBS Lett. (1990) 276:172–4. 10.1016/0014-5793(90)80535-Q 1702393

[71] Jespersen MC Peters B Nielsen M Marcatili P. BepiPred-2.0: improving sequence-based B-cell epitope prediction using conformational epitopes. Nucl Acids Res. (2017) 45:W24–29. 10.1093/nar/gkx346 28472356 PMC5570230

[72] Andersen PH Nielsen M Lund O. Prediction of residues in discontinuous B-cell epitopes using protein 3D structures. Protein Sci. (2006) 15:2558–67. 10.1110/ps.062405906 17001032 PMC2242418

[73] Ponomarenko J Bui H-H Li W Fusseder N Bourne PE Sette A. ElliPro: a new structure-based tool for the prediction of antibody epitopes. BMC Bioinform. (2008) 9:514. 10.1186/1471-2105-9-514 19055730 PMC2607291

[74] Jumper J Evans R Pritzel A Green T Figurnov M Ronneberger O. Highly accurate protein structure prediction with AlphaFold. Nature. (2021) 596:583–9. 10.1038/s41586-021-03819-2 34265844 PMC8371605

[75] Barbier M Wirth T. The evolutionary history, demography, and spread of the *Mycobacterium tuberculosis* complex. Microbiol Spectr. (2016) 4:8. 10.1128/microbiolspec.TBTB2-0008-2016 27726798

[76] Michelet L Richomme C Réveillaud E Cruz KD Moyen J-L Boschiroli ML. *Mycobacterium microti* infection in red foxes in France. Microorganisms. (2021) 9:1257. 10.3390/microorganisms9061257 34207760 PMC8227042

[77] Pérez de Val B Sanz A Soler M Allepuz A Michelet L Boschiroli ML. *Mycobacterium microti* infection in free-ranging wild boar, Spain, 2017–2019. Emerg Infect Dis. (2019) 25:2152–4. 10.3201/eid2511.190746 31625855 PMC6810215

[78] Crispell J Zadoks RN Harris SR Paterson B Collins DM de-Lisle GW. Using whole genome sequencing to investigate transmission in a multi-host system: *Bovine tuberculosis* in New Zealand. BMC Genom. (2017) 18:180. 10.1186/s12864-017-3569-x 28209138 PMC5314462

[79] Merker M Kohl TA Niemann S Supply P. The evolution of strain typing in the *Mycobacterium tuberculosis* complex. In:Gagneux S, editor. Strain Variation in the Mycobacterium tuberculosis Complex: Its Role in Biology, Epidemiology and Control, Advances in Experimental Medicine and Biology. Cham: Springer (2017). p. 43–78. 10.1007/978-3-319-64371-7_3 29116629

[80] Akhmetova A Guerrero J McAdam P Salvador LCM Crispell J Lavery J. Genomic epidemiology of *Mycobacterium bovis* infection in sympatric badger and cattle populations in Northern Ireland. Microb Genom. (2023) 9:mgen001023. 10.1099/mgen.0.001023 37227264 PMC10272874

[81] Duault H Michelet L Boschiroli M-L Durand B Canini L. A Bayesian evolutionary model towards understanding wildlife contribution to F4-family *Mycobacterium bovis* transmission in the South-West of France. Vet Res. (2022) 53:28. 10.1186/s13567-022-01044-x 35366933 PMC8976416

[82] Perea C Ciaravino G Stuber T Thacker TC Robbe-Austerman S Allepuz A. Whole-genome SNP analysis identifies putative *Mycobacterium bovis* transmission clusters in livestock and wildlife in Catalonia, Spain. Microorganisms. (2021) 9:1629. 10.3390/microorganisms9081629 34442709 PMC8401651

[83] van Tonder AJ Thornton MJ Conlan AJK Jolley KA Goolding L Mitchell AP. Inferring *Mycobacterium bovis* transmission between cattle and badgers using isolates from the Randomised Badger Culling Trial. PLoS Pathog. (2021) 17:e1010075. 10.1371/journal.ppat.1010075 34843579 PMC8659364

[84] Dippenaar A Parsons SDC Sampson SL van der Merwe RG Drewe JA Abdallah AM. Whole genome sequence analysis of *Mycobacterium suricattae*. Tuberculosis. (2015) 95:682–8. 10.1016/j.tube.2015.10.001 26542221

[85] Cerezo-Cortés M Rodríguez-Castillo J Hernández-Pando R Murcia M. Circulation of *M. tuberculosis* Beijing genotype in Latin America and the Caribbean. Pathog Glob Health. (2019) 113:336–51. 10.1080/20477724.2019.1710066 31903874 PMC7006823

[86] Zheng H Lu L Wang B Pu S Zhang X Zhu G. Genetic basis of virulence attenuation revealed by comparative genomic analysis of *Mycobacterium tuberculosis* strain H37Ra versus H37Rv. PLoS ONE. (2008) 3:e2375. 10.1371/journal.pone.0002375 18584054 PMC2440308

[87] Stavrum R Valvatne H Bø TH Jonassen I Hinds J Butcher PD. Genomic diversity among Beijing and non-Beijing *Mycobacterium tuberculosis* isolates from Myanmar. PLoS ONE. (2008) 3:e1973. 10.1371/journal.pone.0001973 18398483 PMC2276860

[88] Gordon SV Brosch R Billault A Garnier T Eiglmeier K Cole ST. Identification of variable regions in the genomes of tubercle bacilli using bacterial artificial chromosome arrays. Mol Microbiol. (1999) 32:643–55. 10.1046/j.1365-2958.1999.01383.x 10320585

[89] Gómez-González PJ Campino S Phelan JE Clark TG. Portable sequencing of *Mycobacterium tuberculosis* for clinical and epidemiological applications. Brief Bioinform. (2022) 23:bbac256. 10.1093/bib/bbac256 35894606 PMC9487601

[90] Coscolla M Gagneux S Menardo F Loiseau C Ruiz P Borrell S. Phylogenomics of *Mycobacterium africanum* reveals a new lineage and a complex evolutionary history. Microbiol Genom. (2021) 7:e000477. 10.1099/mgen.0.000477 33555243 PMC8208692

[91] Tsolaki AG Gagneux S Pym AS Goguet de la Salmoniere YOL Kreiswirth BN Van Soolingen D. Genomic deletions classify the Beijing/W strains as a distinct genetic lineage of *Mycobacterium tuberculosis*. J Clin Microbiol. (2005) 43:3185–91. 10.1128/JCM.43.7.3185-3191.2005 16000433 PMC1169157

[92] Sampson SL Warren RM Richardson M Victor TC Jordaan AM. IS6110-mediated deletion polymorphism in the direct repeat region of clinical isolates of *Mycobacterium tuberculosis*. J Bacteriol. (2003) 185:2856–66. 10.1128/JB.185.9.2856-2866.2003 12700265 PMC154393

[93] Al-Khodari NY Al-Attiyah R Mustafa AS. Identification, diagnostic potential, and natural expression of immunodominant seroreactive peptides encoded by five *Mycobacterium tuberculosis*-specific genomic regions. Clin Vac Immunol. (2011) 18:477–82. 10.1128/CVI.00405-10 21177915 PMC3067373

[94] Lyashchenko KP Grandison A Keskinen K Sikar-Gang A Lambotte P Esfandiari J. Identification of novel antigens recognized by serum antibodies in *Bovine tuberculosis*. Clin Vac Immunol. (2017) 24:e00259–17. 10.1128/CVI.00259-17 28978510 PMC5717178

[95] Mukherjee P Dutta M Datta P Dasgupta A Pradhan R Pradhan M. The RD1-encoded antigen Rv3872 of *Mycobacterium tuberculosis* as a potential candidate for serodiagnosis of tuberculosis. Clin Microbiol Infect. (2007) 13:146–52. 10.1111/j.1469-0691.2006.01660.x 17328726

[96] Xu J-N Chen J-P Chen D-L. Serodiagnosis efficacy and immunogenicity of the fusion protein of *Mycobacterium tuberculosis* composed of the 10-kilodalton culture filtrate protein, ESAT-6, and the extracellular domain fragment of PPE68. Clin Vac Immunol. (2012) 19:536–44. 10.1128/CVI.05708-11 22357648 PMC3318282

[97] Ren N JinLi J Zhou X Wang J Ge P Khan FA. Identification of new diagnostic biomarkers for *Mycobacterium tuberculosis* and the potential application in the serodiagnosis of human tuberculosis. Microb Biotechnol. (2018) 11:893–904. 10.1111/1751-7915.13291 29952084 PMC6116745

[98] D'Souza C Kishore U Tsolaki AG. The PE-PPE family of *Mycobacterium tuberculosis:* proteins in disguise. Immunobiology. (2023) 228:152321. 10.1016/j.imbio.2022.152321 36805109

[99] Karboul A Mazza A. Frequent homologous recombination events in *Mycobacterium tuberculosis* PE/PPE multigene families: potential role in antigenic variability. J Bacteriol. (2008) 190:7838–46. 10.1128/JB.00827-08 18820012 PMC2583619

[100] Moens C Filée P Boes A Alie C Dufrasne F André E. Identification of new *Mycobacterium bovis* antigens and development of a multiplexed serological bead-immunoassay for the diagnosis of *Bovine tuberculosis* in cattle. PLoS ONE. (2023) 18:e0292590. 10.1371/journal.pone.0292590 37812634 PMC10561873

[101] Clifford V He Y Zufferey C Connell T Curtis N. Interferon gamma release assays for monitoring the response to treatment for tuberculosis: a systematic review. Tuberculosis. (2015) 95:639–50. 10.1016/j.tube.2015.07.002 26515270

[102] Nuñez-Garcia J Downs SH Parry JE Abernethy DA Broughan JM Cameron AR. Meta-analyses of the sensitivity and specificity of ante-mortem and post-mortem diagnostic tests for bovine tuberculosis in the UK and Ireland. Prev Vet Med. (2018) 153:94–107. 10.1016/j.prevetmed.2017.02.017 28347519

[103] Greenaway C Lienhardt C Adegbola R Brusasca P McAdam K Menzies D. Humoral response to *Mycobacterium tuberculosis* antigens in patients with tuberculosis in the Gambia. Int J Tuberc Lung Dis. (2005) 9:1112–9. 16229222

[104] Rosenkrands I Aagaard C Weldingh K Brock I Dziegiel MH Singh M. Identification of Rv0222 from RD4 as a novel serodiagnostic target for tuberculosis. Tuberculosis. (2008) 88:335–43. 10.1016/j.tube.2007.12.001 18243798

[105] Baassi L Sadki K Seghrouchni F Contini S Cherki W Nagelkerke N. Evaluation of a multi-antigen test based on B-cell epitope peptides for the serodiagnosis of pulmonary tuberculosis. Int J Tuberc Lung Dis. (2009) 13:848–54. 19555534

[106] Sun P Ju H Liu Z Ning Q Zhang J Zhao X. Bioinformatics resources and tools for conformational B-cell epitope prediction. Comput Math Methods Med. (2013) 2013:e943636. 10.1155/2013/943636 23970944 PMC3736542

[107] Zargaran FN Akya A Rezaeian S Ghadiri K Lorestani RC Madanchi H. B cell epitopes of four fimbriae antigens of *Klebsiella pneumoniae*: a comprehensive *in silico* study for vaccine development. Int J Pept Res Ther. (2021) 27:875–86. 10.1007/s10989-020-10134-3 33250677 PMC7684152

[108] Potocnakova L Bhide M Pulzova LB. An introduction to B-cell epitope mapping and *in silico* epitope prediction. J Immunol Res. (2016) 2016:e6760830. 10.1155/2016/6760830 28127568 PMC5227168

[109] Roupie V Alonso-Velasco E Van Der Heyden S Holbert S Duytschaever L Berthon P. Evaluation of mycobacteria-specific gamma interferon and antibody responses before and after a single intradermal skin test in cattle naturally exposed to *M. avium* subsp paratuberculosis and experimentally infected with *M. bovis*. Vet Immunol Immunopathol. (2018) 196:35–47. 10.1016/j.vetimm.2017.12.007 29695323

[110] Aagaard C Brock I Olsen A Ottenhoff THM Weldingh K Andersen P. Mapping immune reactivity toward Rv2653 and Rv2654: two novel low-molecular-mass antigens found specifically in the *Mycobacterium tuberculosis* complex. J Infect Dis. (2004) 189:812–9. 10.1086/381679 14976597

[111] Moens C Saegerman C Fretin D Marché S. Field evaluation of two commercial serological assays for detecting bovine tuberculosis. Res Vet Sci. (2023) 159:125–32. 10.1016/j.rvsc.2023.04.004 37126914

