# Supplementary figures and images for: Genomic comparison between Mycobacterium bovis and Mycobacterium microti and in silico analysis of peptide-based biomarkers for serodiagnosis
Source: Front Vet Sci. 2024 Sep 20;11:1446930. doi: 10.3389/fvets.2024.1446930 (PMC11449866; doi:10.3389/fvets.2024.1446930)

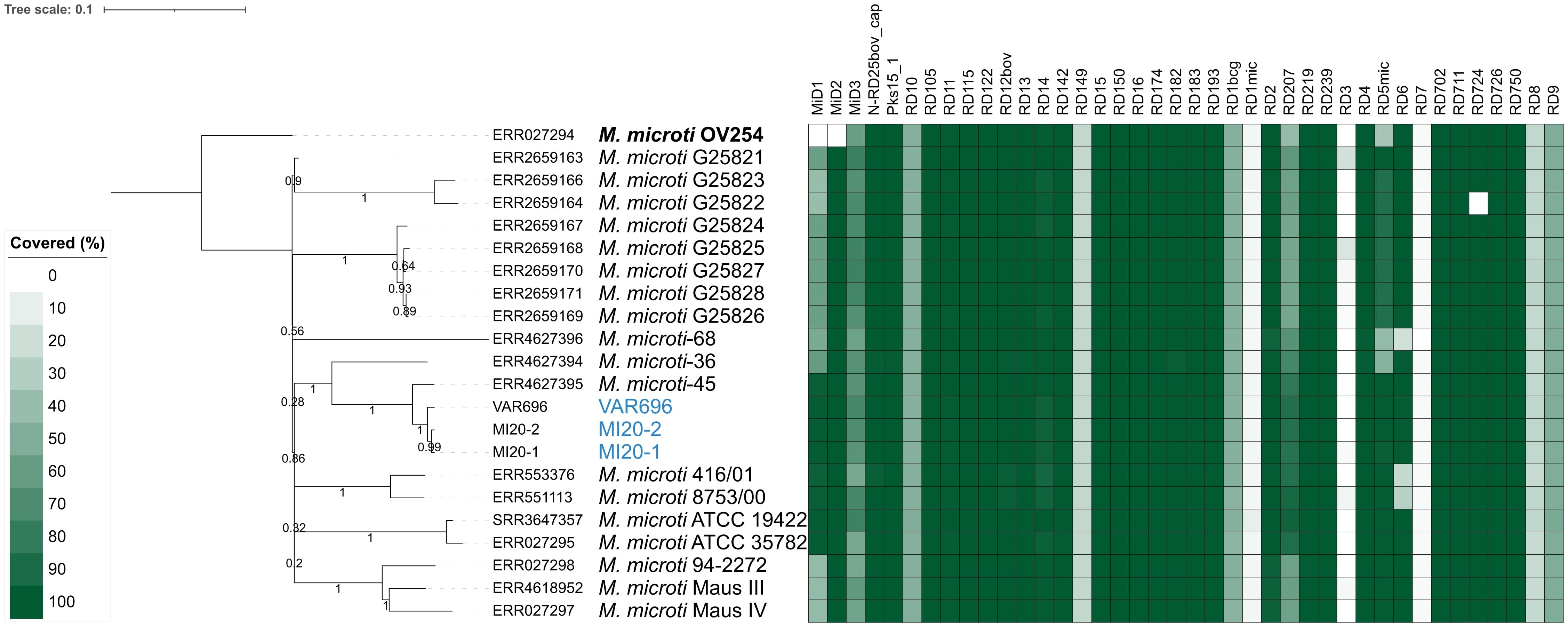

Supplement: Supplementary Figure 1 — Presence of RDs and MiDs regions in the M. microti datasets. This figure shows the percentage of the corresponding RDs and MiDs regions that were covered by the read mapping-based approach for each dataset. [file Image_1.jpeg]

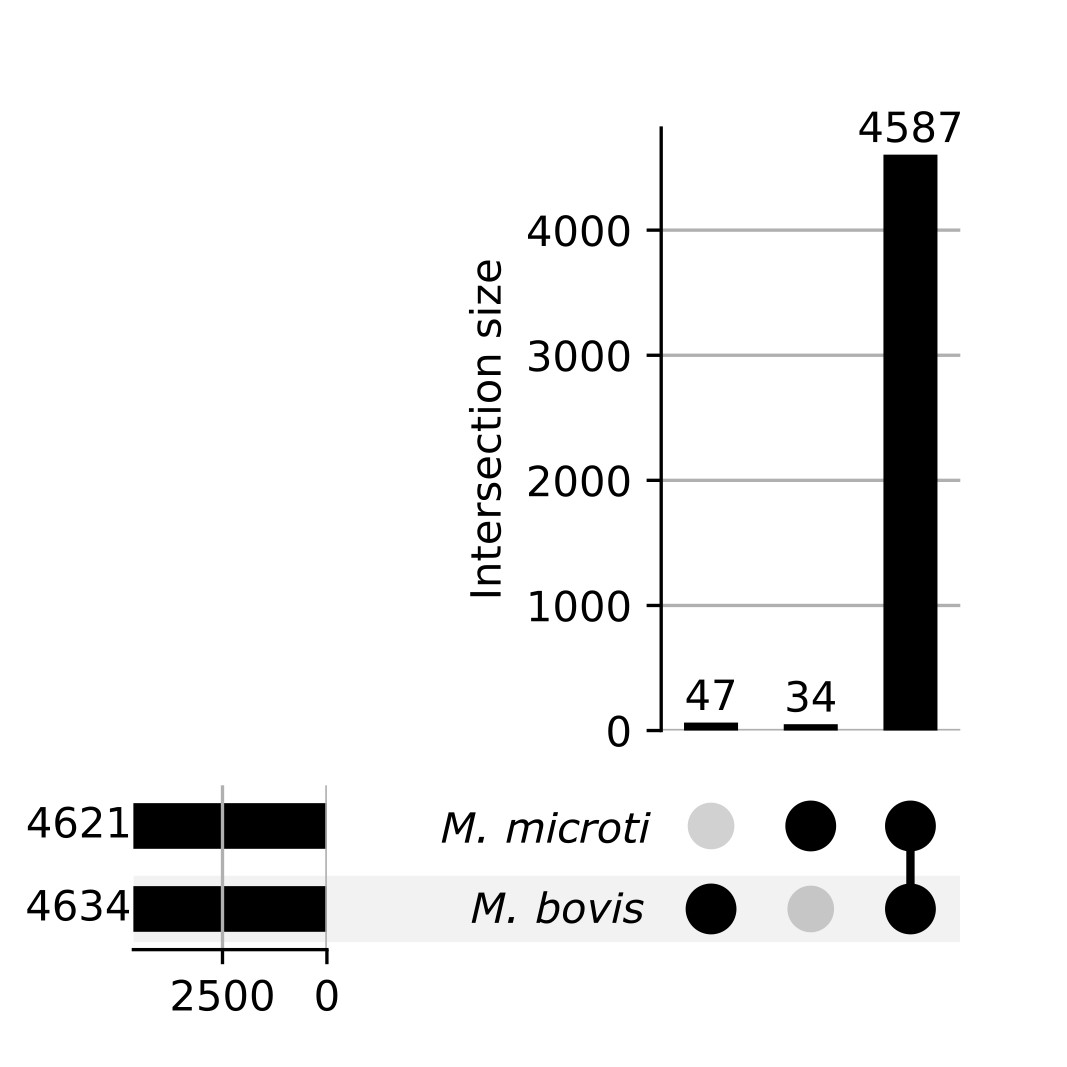

Supplement: Supplementary Figure 2 — Results of the pan-genome analysis, showing the number of genes detected exclusively in all M. microti and all virulent M. bovis strains. Note that all 22 M. microti strains and the two virulent M. bovis strains (AF2122/97 and MB3601) were considered. The horizontal bars indicate the total number of genes detected in the corresponding species by the pan-genome analysis. The vertical lines indicate the size of the overlap as indicated by the dots in membership matrix. [file Image_2.jpeg]
